# New Insights into the Cyclic Di-adenosine Monophosphate (c-di-AMP) Degradation Pathway and the Requirement of the Cyclic Dinucleotide for Acid Stress Resistance in Staphylococcus aureus*

**DOI:** 10.1074/jbc.M116.747709

**Published:** 2016-11-10

**Authors:** Lisa Bowman, Merve S. Zeden, Christopher F. Schuster, Volkhard Kaever, Angelika Gründling

**Affiliations:** From the ‡ Section of Microbiology and Medical Research Council Centre for Molecular Bacteriology and Infection, Imperial College London, London SW7 2AZ, United Kingdom and; the § Research Core Unit Metabolomics, Hannover Medical School, Hannover D-306625, Germany

**Keywords:** bacterial signal transduction, pH regulation, phosphodiesterases, Staphylococcus aureus (S. aureus), stress, YbbR

## Abstract

Nucleotide signaling networks are key to facilitate alterations in gene expression, protein function, and enzyme activity in response to diverse stimuli. Cyclic di-adenosine monophosphate (c-di-AMP) is an important secondary messenger molecule produced by the human pathogen *Staphylococcus aureus* and is involved in regulating a number of physiological processes including potassium transport. *S. aureus* must ensure tight control over its cellular levels as both high levels of the dinucleotide and its absence result in a number of detrimental phenotypes. Here we show that in addition to the membrane-bound Asp-His-His and Asp-His-His-associated (DHH/DHHA1) domain-containing phosphodiesterase (PDE) GdpP, *S. aureus* produces a second cytoplasmic DHH/DHHA1 PDE Pde2. Although capable of hydrolyzing c-di-AMP, Pde2 preferentially converts linear 5′-phosphadenylyl-adenosine (pApA) to AMP. Using a *pde2* mutant strain, pApA was detected for the first time in *S. aureus,* leading us to speculate that this dinucleotide may have a regulatory role under certain conditions. Moreover, pApA is involved in a feedback inhibition loop that limits GdpP-dependent c-di-AMP hydrolysis. Another protein linked to the regulation of c-di-AMP levels in bacteria is the predicted regulator protein YbbR. Here, it is shown that a *ybbR* mutant *S. aureus* strain has increased acid sensitivity that can be bypassed by the acquisition of mutations in a number of genes, including the gene coding for the diadenylate cyclase DacA. We further show that c-di-AMP levels are slightly elevated in the *ybbR* suppressor strains tested as compared with the wild-type strain. With this, we not only identified a new role for YbbR in acid stress resistance in *S. aureus* but also provide further insight into how c-di-AMP levels impact acid tolerance in this organism.

## Introduction

The Gram-positive bacterium *Staphylococcus aureus* is persistently carried by ∼30% of the human population (1). This colonization is often asymptomatic, but *S. aureus* can become pathogenic and cause various diseases ranging from skin and soft tissue infections to pneumonia and bacteremia (2). This microorganism has also developed resistance to many antibiotics, which has reduced the number of therapeutic choices available (3, 4). Although methicillin-resistant *S. aureus* (MRSA)^4^ is mostly associated with the hospital setting, community-associated MRSA (CA-MRSA) strains have spread rapidly in some countries (3).

Bacteria have a remarkable ability to readily adapt to fluctuating environmental conditions and cope with periods of stress. Signal transduction pathways are crucial to achieving this flexibility, where stimuli are sensed and signals relayed within the cell to trigger the induction of an appropriate response. Signaling nucleotides, functioning as secondary messenger molecules, are known to regulate a diverse array of cellular networks in all forms of life. Cyclic adenosine monophosphate (cAMP) and cyclic di-guanine monophosphate (c-di-GMP) are among the best studied of these molecules and regulate a number of physiological processes including carbon catabolism and the switch between a sessile and motile lifestyle, respectively (5). The functions of the stringent response alarmones guanosine tetraphosphate and pentaphosphate ((p)ppGpp) have also been extensively investigated. These nucleotides are produced under various stress conditions including nutrient limitation. They act to decrease the expression of genes required for active growth and increase the expression of amino acid synthesis and transporter genes (6). Although more recently discovered, an impressive effort has been made to elucidate the function of cyclic di-adenosine monophosphate (c-di-AMP) in bacteria.

The signaling molecule c-di-AMP is predominantly produced by Gram-positive bacteria belonging to the Firmicutes and Actinobacteria phyla but can also be synthesized by some Gram-negative bacteria (7–14). c-di-AMP is synthesized from two molecules of ATP via a condensation reaction requiring the activity of a diadenylate cyclase (DAC), and hydrolyzed by a phosphodiesterase (PDE) into 5′-phosphadenylyl-adenosine (pApA), or two molecules of AMP (7, 8, 10, 15–17). *S. aureus* produces a single diadenylate cyclase enzyme called DacA, which is encoded by the first gene in the *dacA-ybbR-glmM* operon (7, 11). The third gene of this operon encodes the phosphoglucosamine mutase GlmM, a cytoplasmic enzyme that converts glucosamine-6-phosphate into glucosamine-1-phosphate (18–20). This product is subsequently used for the production of an early peptidoglycan synthesis intermediate. The second gene in the operon, *ybbR*, encodes a predicted membrane protein that has been shown to interact with, and influence the activity of DacA (21–23). However, there are conflicting reports as to whether YbbR stimulates or inhibits the activity of the cyclase. Therefore, a conclusive role for YbbR is still lacking.

GdpP (also named YybT or Pde1) was the first c-di-AMP-hydrolyzing enzyme identified in *Bacillus subtilis* (15). GdpP contains two N-terminal transmembrane helices followed by a cytoplasmic Per-Arnt-Sim (PAS) domain, a modified GGDEF domain, and a DHH/DHHA1 domain, which possesses phosphodiesterase activity (15). GdpP homologs have since been discovered in a range of microorganisms including *Streptococcus pneumoniae*, *Listeria monocytogenes* and *S. aureus*, and function to specifically degrade c-di-AMP into pApA (7, 17, 24, 25). The absence of GdpP in *S. aureus* leads to an ∼10-fold increase in cellular c-di-AMP levels, a reduced bacterial cell size, and production of a peptidoglycan polymer with increased cross-linking (7). Further studies have also correlated levels of c-di-AMP in *S. aureus* with resistance to β-lactam antibiotics (7, 26–28).

A cytoplasmic protein containing a stand-alone DHH/DHHA1 domain, but lacking the sensory domains found in GdpP, has also been associated with the metabolism of c-di-AMP (14, 24, 29). In *Borrelia burgdorferi* the DHH/DHHA1 domain containing protein DhhP has been described as a c-di-AMP PDE and essential for growth (14). *S. pneumoniae* produces a homolog of DhhP named Pde2, which can hydrolyze c-di-AMP and pApA as well as 3′-phosphoadenosine-5′-phosphate (pAp) to AMP and is essential for pathogenesis (24, 29). The *Mycobacterium tuberculosis* and *Mycobacterium smegmatis* homologs, MtbPDE and MtPDE, respectively, have been shown to degrade both c-di-AMP and pApA (30–33). A third class of c-di-AMP PDE found in c-di-AMP-producing microorganisms is an HD-domain containing enzyme (17, 34). Similarly to GdpP, this PDE is an integral membrane protein and degrades c-di-AMP into pApA (17, 34).

In this study we further investigated the c-di-AMP metabolism in *S. aureus* by examining the function of the DHH/DHHA1 PDE Pde2 and the membrane protein YbbR. We show that the *S. aureus* PDE Pde2 preferentially hydrolyzes pApA over c-di-AMP, leading in its absence to an accumulation of pApA. We also show that deleting *ybbR* in *S. aureus* does not significantly affect c-di-AMP production or growth under standard growth conditions. However, its absence leads to increased acid sensitivity, which can be compensated by the production of an altered DacA variant.

## Results

### 

#### 

##### An S. aureus pde2 Mutant Strain Displays a Growth Defect in the Early Growth Phase

**FIGURE 1.**
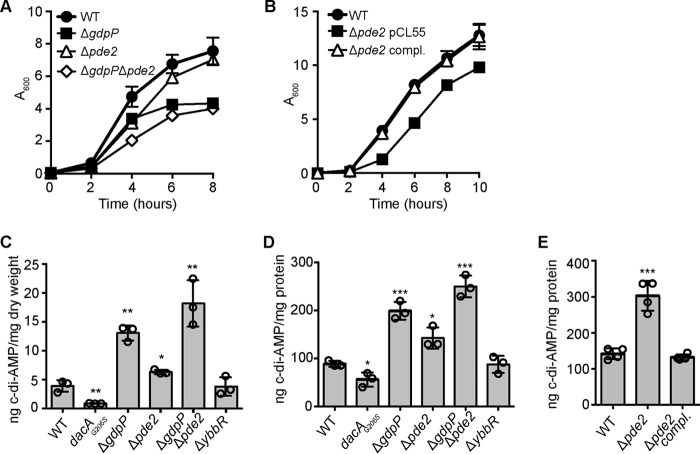
**An *S. aureus pde2* mutant has a slight growth defect and increased cellular c-di-AMP levels.** *A* and *B*, bacterial growth curve. *A*, cultures of WT LAC* and the indicated mutant strains were diluted to an *A*_600_ of 0.05, grown to an *A*_600_ of 1, and back-diluted again to an *A*_600_ of 0.05 with this time point set to time = 0. Growth was subsequently monitored over an 8-h period, and average *A*_600_ readings and standard deviations from three independent experiments are plotted. *B*, overnight culture of LAC* (WT) and the *pde2* mutant strain containing the vector pCL55 as well as the *pde2* complementation strain were diluted to an *A*_600_ of 0.01, and growth was monitored over 10 h. Average *A*_600_ readings and standard deviations from three independent experiments are plotted. *C*, determination of intracellular c-di-AMP levels by LC-MS/MS. LAC* (WT) and the indicated mutants were grown overnight in TSB medium, cell extracts were prepared, and c-di-AMP levels were determined by LC-MS/MS. The nucleotide levels are reported as ng of c-di-AMP/mg cell dry weight, and the average values and standard deviations from three independent experiments are plotted. *D,* determination of intracellular c-di-AMP levels by ELISA. The same strains as in *C* were grown overnight in TSB medium, cell extracts were prepared, and c-di-AMP levels were determined by ELISA. The nucleotide levels are reported as ng of c-di-AMP/mg of protein, and the average values and standard deviations from three independent experiments are plotted. *E*, determination of intracellular c-di-AMP levels by ELISA. Cell extracts were prepared from LAC* (WT), and the *pde2* mutant strain containing the vector pCL55 as well as the *pde2* complementation strain and c-di-AMP levels were determined by ELISA. The nucleotide levels are reported as ng of c-di-AMP/mg protein, and the average values and standard deviations from four independent experiments are plotted. Statistical analysis was performed using an unpaired Student's *t* test where one asterisk (*), two asterisks (**), or three asterisks (***) indicate differences between WT and mutant strains with *p* values of <0.05, <0.01, or <0.001, respectively.

Although HD domain-containing PDEs are not found in *Staphylococcus*, a stand-alone DHH/DHHA1 domain protein and Pde2 ortholog is present in *S. aureus*. This protein corresponds to SAUSA300_1650 in *S. aureus* strain FRP5737 and will be from here on out referred to as Pde2. To assess the function of Pde2 in *S. aureus,* strain LAC*Δ*pde2*::*erm* was constructed in which the *pde2* gene was replaced by an erythromycin marker. When grown in tryptic soya broth (TSB) medium, LAC*Δ*pde2*::*erm* exhibited a growth defect in the early stages of growth similar to that observed for the *gdpP* mutant strain LAC*Δ*gdpP*::*kan* (Fig. 1*A*). In contrast to the *gdpP* mutant, LAC*Δ*pde2*::*erm* reached a similar density as the WT LAC* control culture at later time points (Fig. 1*A*). This growth defect could be complemented by expressing *pde2* from its native promoter from the single site integration vector in strain LAC*Δ*pde2*::*erm* pCL55-*pde2* (Fig. 1*B*). Strain LAC*Δ*gdpP::kan*Δ*pde2*::*erm*, deleted for both *gdpP* and *pde2*, exhibited a greater growth defect as compared with the single mutants at the early time points, with the culture reaching a similar density as the *gdpP* mutant strain at the 8-h time point (Fig. 1*A*). This growth analysis revealed that the *pde2* and *gdpP* mutants have distinct growth phenotypes, and the additive growth defect observed in the double mutant suggests that Pde2 might also be involved in the c-di-AMP metabolism of *S. aureus*.

##### Deletion of pde2 in S. aureus Leads to an Increase in Cellular c-di-AMP Levels and Increased Oxacillin Resistance

To directly assess the contribution of Pde2 to the c-di-AMP metabolism in *S. aureus,* cytosolic extracts were prepared from WT LAC*, the isogenic *pde2, gdpP* single mutants, and the *gdpP/pde2* double mutant strains. As an additional control, strain LAC*dacA_G206S_ was constructed, which produces a DacA variant with a glycine-to-serine change at amino acid residue 206. *S. aureus* strains containing this *dacA*_G206S_ allele have been reported to produce lower amounts of c-di-AMP (26). Next, the cellular c-di-AMP concentration was determined by LC-MS/MS as well as by an ELISA assay. As expected, decreased or increased amounts of c-di-AMP were found in extracts derived from the *dacA*_G206S_ and *gdpP* mutant strains, respectively, when compared with the WT LAC* control strain (Fig. 1, *C* and *D*). Using both c-di-AMP detection methods (LC-MS/MS and ELISA), a slight but significant increase in the cellular c-di-AMP concentration was also observed for the *pde2* mutant when compared with WT LAC*, with the levels in the double mutant found to be even greater than in the *gdpP* single mutant (Fig. 1, *C* and *D*). Finally, as assessed by ELISA, the c-di-AMP levels were restored to wild-type levels in the *pde*2 complementation strain LAC*Δ*pde2*::*erm* pCL55-*pde2* (Fig. 1*E*). Altogether, these data suggest that Pde2 contributes to the c-di-AMP metabolism in *S. aureus*. A correlation between c-di-AMP levels and oxacillin resistance of MRSA strains has been observed in previous studies where strains with high and low dinucleotide levels exhibit increased and decreased resistance, respectively (7, 26). To assess the contribution of Pde2 to oxacillin resistance, M.I.C. Evaluator^TM^ strips were used to determine the susceptibility of the different strains to this antibiotic. The oxacillin MIC for WT LAC* was 4 μg/ml, and as expected, the *gdpP* and *dacA*_G206S_ mutants showed higher and lower resistances, respectively, with MICs of >256 μg/ml and 0.12 μg/ml. Similar to the *gdpP* mutant, the *pde2* single and *gdpP/pde2* double mutant strains also displayed an increased resistance to oxacillin with MICs of >256 μg/ml. It is, however, of note that although growth was observed along all concentrations of the antibiotic strip for the *gdpP* and *pde2* single mutant strains, a halo, and area of reduced growth, was visible at concentrations above 4 μg/ml but not visible for the *gdpP/pde2* double mutant strain (data now shown). Taken together, these results indicate that Pde2 contributes to oxacillin resistance and the metabolism of c-di-AMP in *S. aureus*.

##### S. aureus Pde2 Preferentially Hydrolyzes pApA over c-di-AMP

**FIGURE 2.**
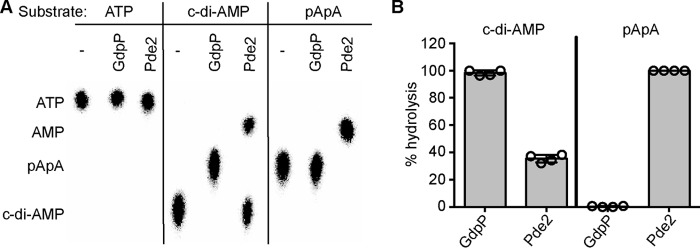
**Pde2 degrades both c-di-AMP and pApA.** *A*, GdpP and Pde2 enzyme activity assays. 100 μm ATP, c-di-AMP, or pApA spiked with a low nm concentration of the respective radiolabeled nucleotides were incubated for 2 h at 37 °C in the absence of enzyme or with 1 μm purified GdpP or Pde2. The reaction products were separated by TLC, and radioactive signals were detected using a phosphorimager. Substrate nucleotides are shown on *top*, and nucleotide products are listed along the *left* of the figure. A representative image from four independent experiments is shown. *B*, quantification of GdpP and Pde2 nucleotide hydrolysis activity. The % c-di-AMP or pApA substrate hydrolysis by GdpP and Pde2 was determined by quantification of the radioactive signal of TLC plates shown in *A*, and the average value and standard deviations from four experiments are plotted.

**FIGURE 3.**
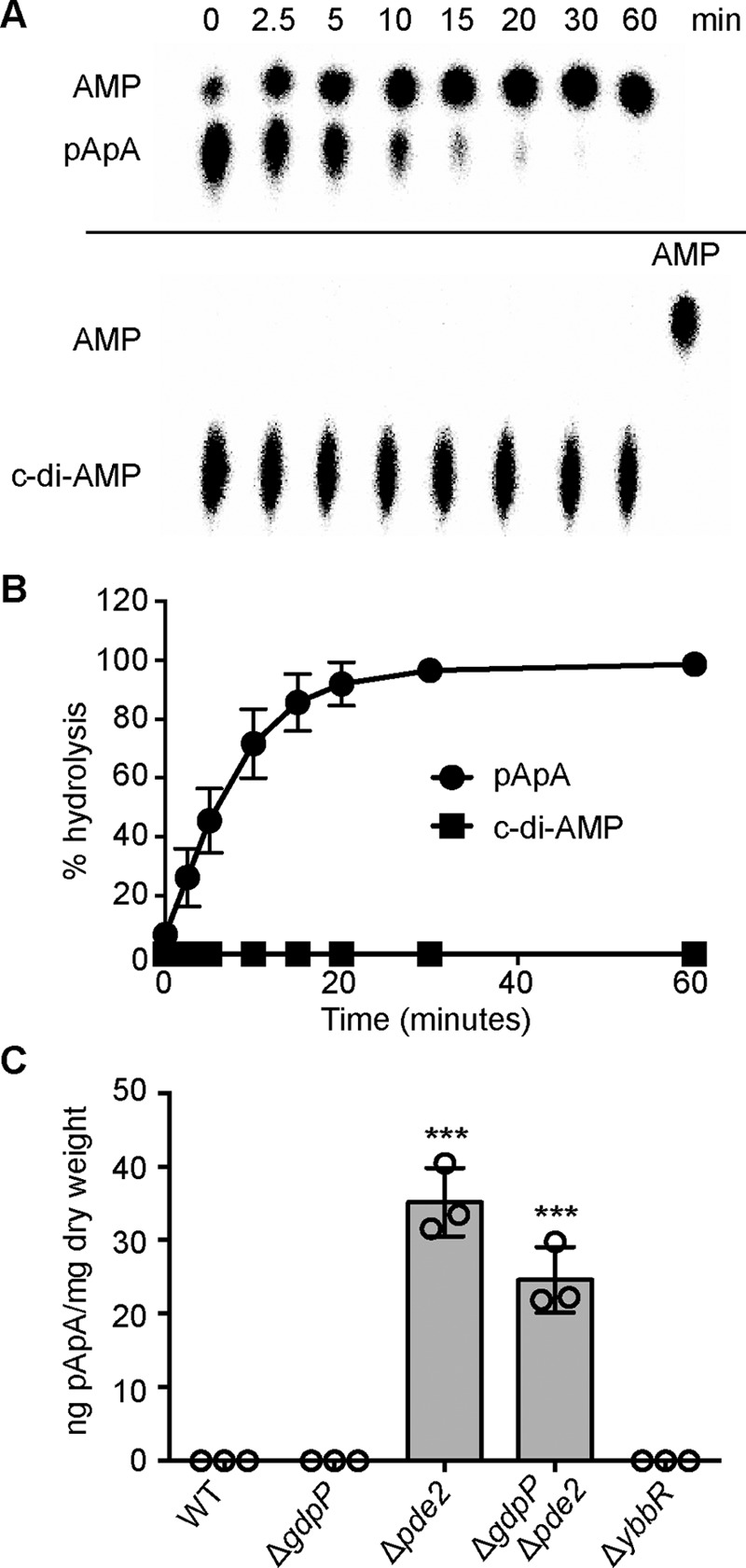
**Pde2 preferably hydrolyzes pApA over c-di-AMP and its deletion leads to an accumulation of pApA.** *A*, Pde2 enzyme activity time course experiment. 200 μm pApA or c-di-AMP spiked with a small concentration of the respective radiolabeled nucleotide was incubated with 4 nm concentrations of purified Pde2 at 37 °C. Samples were removed at timed intervals and analyzed by TLC as described in Fig. 2. *B*, quantification of c-di-AMP and pApA hydrolysis by Pde2. The radioactive signals of the input nucleotide were quantified, the % c-di-AMP or pApA substrate hydrolysis was calculated, and the average values and standard deviations from six independent experiments are plotted. *C*, determination of intracellular pApA levels by LC-MS/MS. WT LAC* and the indicated mutants were grown overnight in TSB medium, cell extracts were prepared, and pApA levels were determined by LC-MS/MS as described in the “Experimental Procedure” section. The nucleotide levels are reported as ng of pApA/mg cell dry weight, and the average values and standard deviations from three independent experiments are plotted. Statistical analysis was performed using an unpaired Student's *t* test where three asterisks (***) indicate differences between WT and mutant strains with a *p* value of <0.001.

To determine the substrate specificity of the *S. aureus* Pde2 enzyme, an N-terminally His-tagged version of the protein was expressed and purified from *Escherichia coli*, and used in *in vitro* enzyme activity assays. 1 μm recombinant Pde2 was incubated for 2 h with 100 μm ATP, c-di-AMP, or pApA (spiked with nm concentrations of the respective radiolabeled nucleotide), and reaction products were analyzed by TLC. As a control, reactions were also set up with recombinant GdpP, known to specifically cleave c-di-AMP into pApA (7, 35). As expected, GdpP converted c-di-AMP into a single product, pApA, but did not hydrolyze ATP or pApA (Fig. 2). Pde2 was incapable of hydrolyzing ATP, whereas 35% of the input c-di-AMP and 100% of the input pApA was hydrolyzed and, based on analogy with other Pde2 enzymes, likely converted to AMP (Fig. 2). To confirm that c-di-AMP and pApA were converted to AMP, 100 μm unlabeled c-di-AMP or pApA substrates were incubated with Pde2, and the reaction products were analyzed by LC-UV. This analysis confirmed that Pde2 converted both c-di-AMP and pApA into AMP (data not shown). These initial experiments also indicated that the *S. aureus* Pde2 enzyme preferentially hydrolyzes pApA over c-di-AMP. To investigate this further, a time-course experiment was performed. Complete hydrolysis of 200 μm pApA was observed within 1 h using 4 nm Pde2 (Fig. 3, *A* and *B*). Using this low enzyme concentration, no hydrolysis of c-di-AMP was observed within the 1-h incubation time (Fig. 3, *A* and *B*), suggesting that the native substrate of the *S. aureus* Pde2 enzyme is pApA rather than c-di-AMP.

##### Deletion of pde2 from S. aureus Results in Increased Cellular Levels of pApA

We reasoned that if Pde2 also degrades pApA *in vivo* in *S. aureus*, it is likely that upon deletion of *pde2*, cellular levels of pApA would be elevated. Therefore, cellular extracts were prepared from the WT LAC* strain, *gdpP* and *pde2* single mutants, and the *gdpP/pde2* double mutant strains, and pApA levels were determined by LC-MS/MS analysis. Although pApA was not detectable in WT LAC* or the *gdpP* mutant, the nucleotide was readily detected in the *pde2* deletion strain (Fig. 3*C*). This result supports the *in vitro* enzyme activity assays and suggests that the degradation of pApA is the main cellular function of Pde2 in *S. aureus*. Surprisingly, pApA could also be detected in extracts prepared from the *gdpP*/*pde2* double mutant strain (Fig. 3*C*), suggesting the presence of yet another enzyme that can convert c-di-AMP to pApA in *S. aureus* or the existence of an alternative cellular pathway leading to the generation of pApA.

##### pApA Inhibits GdpP Phosphodiesterase Activity, whereas Pde2 Activity Is Inhibited by ppGpp

**FIGURE 4.**
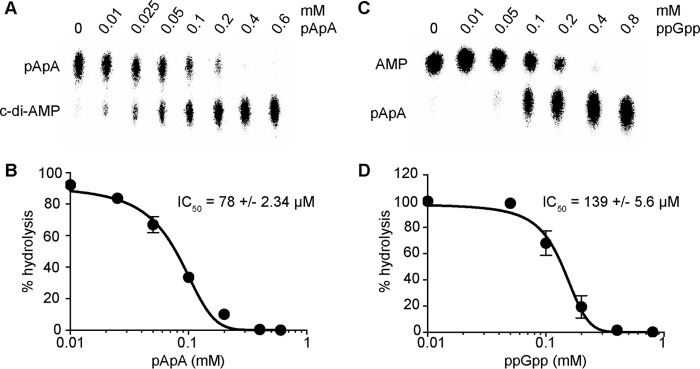
**GdpP phosphodiesterase activity is inhibited by pApA and Pde2 activity by the alarmone ppGpp.** *A*, GdpP enzyme activity in the presence of increasing concentrations of pApA. 50 μm c-di-AMP spiked with a nm amount of radiolabeled c-di-AMP was incubated for 1 h at 37 °C with 0.3 μm GdpP and increasing concentrations of pApA ranging from 0 to 0.6 mm. The reaction products were separated by TLC, and the radioactive signal was detected using a phosphorimager. Experiments were performed in triplicate, and a representative TLC plate is shown. *B*, quantification of c-di-AMP hydrolysis by GdpP in the presence of increasing amounts of pApA. The radioactive signal from *panel A* was quantified, the % c-di-AMP hydrolysis was calculated, and the average values and standard deviations from three independent experiments are plotted. The data were fitted, and the IC_50_ was determined using the dose response inhibition analysis in GraphPad Prism. *C*, Pde2 enzyme activity assay and TLC analysis. 200 μm pApA spiked with radiolabeled pApA was incubated for 1 h at 37 °C with 4 nm Pde2 and increasing concentrations of ppGpp ranging from 0 to 0.8 mm. TLC plates were analyzed as described in *panel A*, and a representative image from three independent experiments is shown. *D*, quantification of pApA hydrolysis by Pde2 in the presence of increasing concentrations of ppGpp. The radioactive signal from *panel C* was quantified, the % pApA hydrolysis was determined, and the average values and standard deviations from three independent experiments are plotted. The data were fitted, and IC_50_ were determined using the dose response inhibition analysis.

The hydrolysis of c-di-AMP by Pde2 is extremely inefficient *in vitro* and may not be of physiological relevance. However, an increase in cellular c-di-AMP levels was detected in a *pde2* mutant *S. aureus* strain along with an increase in pApA levels (Fig. 1, *C–E*, and Fig. 3*C*). It is possible that high levels of pApA may inhibit the activity of GdpP, thereby leading to an elevation of c-di-AMP levels in the cell, even if Pde2 is unable to degrade c-di-AMP *in vivo*. To establish whether pApA can indeed inhibit the PDE activity of GdpP, enzyme reactions were set up with purified GdpP enzyme and 50 μm c-di-AMP (spiked with a nm concentration of radioactive c-di-AMP) and increasing concentrations of pApA. The reactions were incubated for 1 h, and the c-di-AMP hydrolysis activity of GdpP was assessed by TLC. This analysis revealed that pApA could indeed inhibit the GdpP-dependent conversion of c-di-AMP in a dose-dependent manner (Fig. 4*A*), and an IC_50_ value of 78 ± 2.34 μm was established (Fig. 4*B*). These data suggest that an accumulation of pApA in the cell might lead to a negative feedback loop that acts to reduce the rate of c-di-AMP hydrolysis by GdpP in *S. aureus*. This inhibition is similar to that observed for the *Pseudomonas aeruginosa* c-di-GMP PDE RocR by 5′-phosphoguanylyl-guanosine (pGpG) (36, 37).

It has previously been shown that the c-di-AMP PDEs YybT and GdpP of *B. subtilis* and *S. aureus,* respectively, and the HD-domain PDE PgpH of *L. monocytogenes* are inhibited by ppGpp (15, 34, 35). To establish if *S. aureus* Pde2 is also inhibited by ppGpp, degradation of pApA by Pde2 was assessed in the presence of an increasing concentration of ppGpp. This analysis revealed that Pde2 is inhibited by ppGpp in a concentration-dependent manner (Fig. 4*C*), and an IC_50_ of 139 ± 5.6 μm was determined (Fig. 4*D*). These data indicate that the alarmone ppGpp can inhibit the activities of both the membrane-bound c-di-AMP PDE GdpP and the cytoplasmic pApA PDE Pde2 and suggests an importance in limiting c-di-AMP and pApA hydrolysis under nutrient limiting and other stress conditions.

##### Deletion of ybbR Does Not Affect Growth, Oxacillin Resistance, or c-di-AMP Levels in S. aureus under Standard Growth Conditions

**FIGURE 5.**
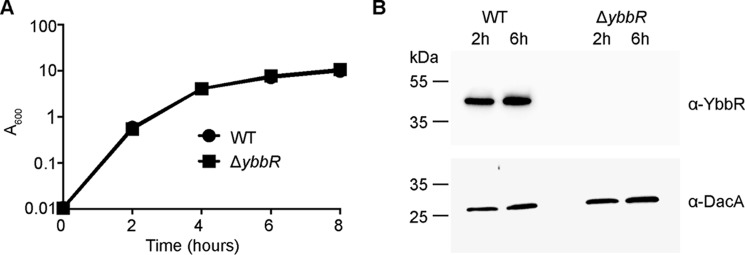
**Deletion of *ybbR* does not affect growth or DacA protein production under standard growth conditions.** *A*, bacterial growth curves. Overnight cultures of WT LAC* and the *ybbR* mutant strain were diluted to an *A*_600_ of 0.01, grown to an *A*_600_ of 1, and again back-diluted to an *A*_600_ of 0.01 (time = 0). Growth was subsequently monitored over an 8-h period, and average *A*_600_ values and standard deviations from three independent experiments are plotted. *B*, detection of DacA and YbbR proteins by Western blotting. Samples were removed at the 2-h and 6-h time points from cultures grown as described in *panel A*, and whole cell protein lysates were prepared. YbbR and DacA proteins were detected by Western blotting using YbbR and DacA specific antibodies. A representative blot of three independent experiments is shown.

Besides Pde2, another protein that has been linked to the metabolism of c-di-AMP in various organisms is YbbR. To investigate the function of YbbR in *S. aureus,* the *ybbR* mutant strain LAC*Δ*ybbR* was generated. The growth of this mutant was compared with that of the WT LAC* strain under standard growth conditions in TSB medium; however, no difference in growth was noted (Fig. 5*A*). To investigate if the deletion of *ybbR* might affect the stability of the DacA cyclase, DacA levels were assessed by Western blotting at the 2- and 6-h time points. However, no significant difference in DacA protein levels was found between the WT and the *ybbR* mutant strain (Fig. 5*B*). Of note, the absence of YbbR in the mutant strain was confirmed by Western blotting analysis using a YbbR-specific antibody (Fig. 5*B*). Next, to establish whether or not YbbR affects c-di-AMP or pApA levels in *S. aureus*, cytoplasmic extracts were prepared from overnight cultures of the WT and LAC*Δ*ybbR* mutant strains and subjected to LC-MS/MS and ELISA analysis. Levels of c-di-AMP, however, remained unchanged in the absence of YbbR (Fig. 1, *C* and *D*), and similar to the WT control strain, no pApA was detected in the mutant (Fig. 3*C*). Consistent with this, the *ybbR* mutant also had an identical oxacillin MIC of 4 μg/ml as observed for the WT LAC* strain. Taken together, these data show that under standard growth conditions, YbbR affects neither DacA protein levels nor the cellular levels of c-di-AMP or pApA or oxacillin resistance in *S. aureus.*

##### The Absence of YbbR Results in Increased Acid Sensitivity

**FIGURE 6.**
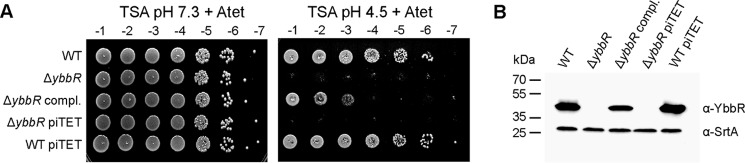
**An *S. aureus ybbR* mutant has increased acid sensitivity.** *A*, bacterial growth on TSA pH 7.3 *versus* TSA pH 4.5 plates. WT LAC* and the *ybbR* mutant as well as the complementation strain LAC*Δ*ybbR* piTET-*ybbR* (Δ*ybbR compl.*) and the empty vector containing control strains Δ*ybbR* piTET and WT piTET were grown overnight in TSB medium. Cultures were then diluted to an *A*_600_ of 5- and 10-fold serial dilutions ranging from 10^−1^ to 10^−7^ prepared and spotted onto TSA pH 7.3 or pH 4.5 plates containing 200 ng/ml Atet. *B*, detection of YbbR by Western blotting. A single colony of the same *S. aureus* strains as described in *panel A* was used to inoculate TSB containing 200 ng/ml Atet, and these starter cultures were incubated for 4 h at 37 °C. Cultures were diluted to an *A*_600_ of 0.05 and grown overnight at 37 °C in TSB 200 ng/ml Atet before preparing whole cell lysates. The YbbR protein and the loading control protein, SrtA, were detected by Western blotting using YbbR- and SrtA-specific antibodies, respectively.

The *S. aureus ybbR* mutant grew identically to the WT LAC* strain in TSB medium (Fig. 5*A*). To identify a growth phenotype and potentially unmask a function for YbbR in *S. aureus*, the WT LAC* strain and *ybbR* mutant were subjected to a phenotypic microarray and grown on several BioLog plates. A significant difference in respiration was observed between the WT and *ybbR* mutant when cultured in rich medium containing urea, and the pH lowered to 4.5. Although it was not possible to obtain information on the exact urea and medium composition within this well, the data suggested a potential difference in the growth between the strains under acid conditions. To investigate this further, serial 10-fold dilutions of LAC* and LAC*Δ*ybbR* cultures were spotted onto standard TSA pH 7.3 and TSA pH 4.5 plates. Interestingly, disruption of *ybbR* led to a decreased resistance to acid stress (Fig. 6*A*). To confirm that this acid sensitivity phenotype is due to the deletion of *ybbR*, the single site integration vector piTET-*ybbR*, which allows for *ybbR* expression from the anhydrotetracyline (Atet)-inducible promoter, was integrated into the *ybbR* mutant strain to produce the complementation strain LAC*Δ*ybbR* piTET-*ybbR* (Δ*ybbR* compl.). The empty piTET vector was also introduced into WT LAC* and LAC*Δ*ybbR* yielding control strains LAC* piTET and LAC*Δ*ybbR* piTET. Production of YbbR in the presence of Atet was confirmed by Western blotting using a YbbR-specific antibody (Fig. 6*B*). It is of note, however, that even upon full induction with 200 ng/ml Atet, the YbbR protein level was reduced in the complementation strain as compared with the LAC* piTET control strain (Fig. 6*B*). The acid sensitivity phenotype of the *ybbR* mutant was partially complemented in the LAC*Δ*ybbR* piTET-*ybbR* strain upon supplementing the medium with Atet (Fig. 6*A*), all together indicating that deletion of *ybbR* does indeed lead to the acid sensitivity phenotype.

##### The Acid Sensitivity of a ybbR Mutant Strain Can Be Bypassed by Compensatory Mutations at Multiple Chromosomal Locations

**FIGURE 7.**
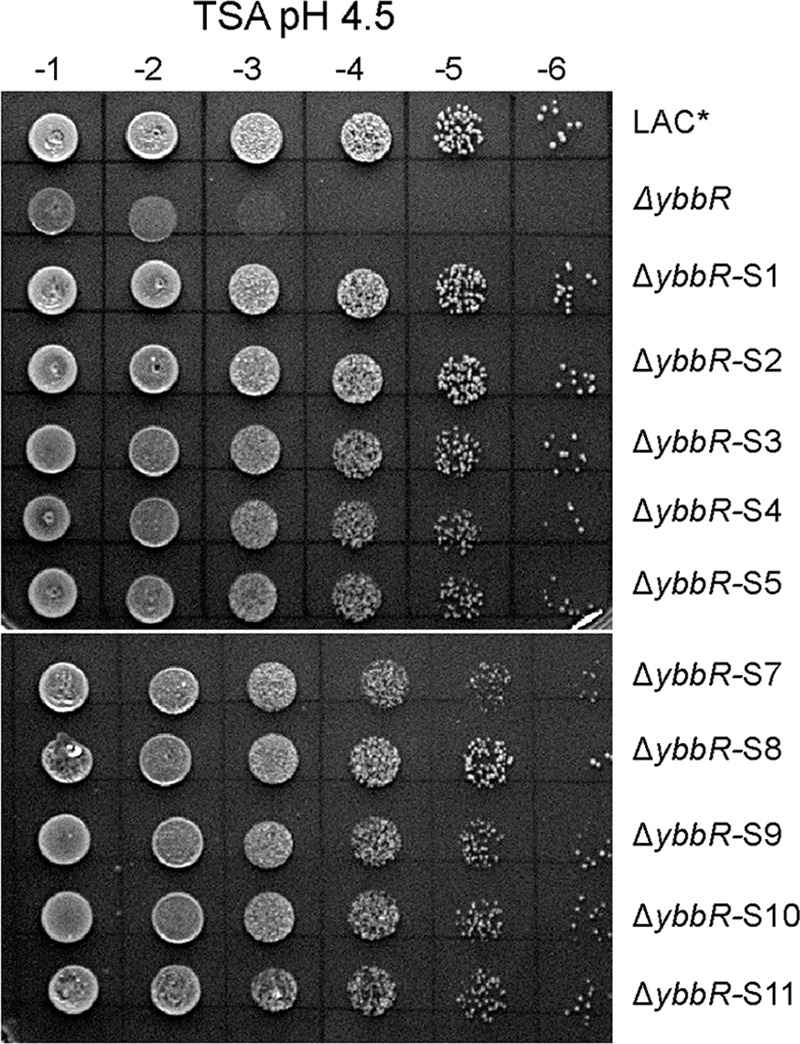
Identification of LAC*Δ*ybbR* suppressor strains with improved acid resistance. WT LAC*, Δ*ybbR*, and Δ*ybbR* suppressor strains (Δ*ybbR*-S1 to S5 and S7 to S11) were grown overnight at 37 °C, cultures were normalized to an *A*_600_ of 5, and 10-fold dilutions ranging from 10^−1^ to 10^−6^ were spotted on TSA pH 4.5 plates.

In an attempt to further understand the contribution of YbbR to acid resistance in *S. aureus*, suppressor strains were raised by plating high cell density aliquots of LAC*Δ*ybbR* cultures on TSA, pH 4.5, plates. Colonies that had grown within 24 h were then further characterized. Increased acid tolerance was confirmed for 10 of these suppressor strains, referred to as LAC*Δ*ybbR-*S1 to S5 and LAC*Δ*ybbR-*S7 to S11, by spotting serial dilutions of cultures on TSA pH 4.5 plates (Fig. 7). As observed for the WT LAC* strain, but in contrast to the *ybbR* mutant strain, a similar number of colonies was obtained for the suppressor strains on both plates (Fig. 7). Whole genome sequencing was then employed to identify potential compensatory mutations in these strains. Good sequence coverage was obtained for eight different LAC*Δ*ybbR* suppressor strains, and their genome sequences were compared with that of the WT LAC* and the LAC*Δ*ybbR* mutant strain. This analysis revealed two high frequency and good quality sequence changes in strain LAC*Δ*ybbR* when compared with strain LAC* (Table 1). These mutations were also present in all suppressor strains, indicating that these mutations must have been acquired during the construction of strain LAC*Δ*ybbR* (Table 1). The different LAC*Δ*ybbR* suppressor strains contained one or two additional high frequency and good quality changes. Interestingly, suppressor strains LAC*Δ*ybbR*-S1 and S2 contained a mutation in *dacA*, resulting in a glycine-to-valine amino acid substitution at amino acid position 78 in the encoded protein. This amino acid is predicted to be located in the third transmembrane helix of the c-di-AMP cyclase DacA. Strains LAC*Δ*ybbR*-S3 and S7 contained a mutation in *proP*, coding for a predicted proline/betaine transporter. LAC*Δ*ybbR*-S4 contained a mutation in *glyA* and *escA* coding for the serine hydroxymethyltransferase GlyA and an ABC transporter ATP-binding protein, respectively. LAC*Δ*ybbR*-S9 contained a mutation in a PilT domain-containing protein, and finally strains LAC*Δ*ybbR*-S10 and S11 contained mutations in a putative membrane-associated zinc metalloprotease (Table 1). In summary, this suppressor mutant analysis indicates that the acid-sensitive phenotype observed in the *ybbR* mutant can be bypassed in multiple ways, one of which likely affects the function of the c-di-AMP cyclase DacA.

**TABLE 1:** Single nucleotide variants and deletions identified in strain LAC*Δ*ybbR* (ANG3301) and eight different LAC*Δ*ybbR* suppressor strains compared to strain LAC* Shown in bold are two sequence changes found in LAC*Δ*ybbR* and all suppressor strains when compared to strain LAC*, indicating that these mutations were acquired during the construction of strain LAC*Δ*ybbR.*

Strain number	Reference position*^a^*	Type*^b^*	Ref.*^c^*	Allele*^d^*	Freq.*^e^*	Av. quality*^f^*	Annotations	AA change*^g^*
**ANG3301 (Δ*ybbR*)**	**1706409**	**SNV**	**G**	**A**	**100**	**37.33**	**SAUSA300_1561, iron transporter of the NRAMP family, MntH**	**A367V**
	**1671814**	**SNV**	**T**	**C**	**85.71**	**35.67**	**SAUSA300_1525, glycyl-tRNA synthetase GlyS**	
ANG3817 (Δ*ybbR-*S1)	2284056	SNV	A	C	95.83	35.3	SAUSA300_2113, c-di-AMP cyclase DacA	V78G
ANG3818 (Δ*ybbR*-S2)	2284056	SNV	A	C	91.67	34.67	SAUSA300_2113, c-di-AMP cyclase DacA	V78G
ANG3855 (Δ*ybbR-*S3)	631381	SNV	G	A	100	31.73	SAUSA300_0558, putative proline/betaine transporter ProP	G26D
ANG3819 (Δ*ybbR-*S4)	2225642	SNV	T	A	96.43	36.78	SAUSA300_2067, serine hydroxymethyl-transferase GlyA	Y61F
	1963962	SNV	C	A	95	35.42	SAUSA300_1786, ABC transporter ATP-binding protein EcsA	Glu-159*
ANG3820 (Δ*ybbR-*S7)	631695	SNV	C	T	100	35.43	SAUSA300_0558, putative proline/betaine transporter ProP	Gln-131*
ANG3822 (Δ*ybbR-*S9)	573037	SNV	T	A	90.9	35.4	SAUSA300_0512, PilT domain-containing protein	Leu-33*
ANG3823 (Δ*ybbR-*S10)	1262490	DEL	A	-	95.45	37	SAUSA300_1155, putative membrane-associated zinc metalloprotease RseP	Lys-239fs
ANG3824 (Δ*ybbR-*S11)	257175	SNV	A	T	100	38.15	SAUSA300_0217, AraC family DNA binding response regulator	D99E
	1262490	DEL	A	-	100	33.06	SAUSA300_1155, putative membrane-associated zinc metalloprotease RseP	K239fs

*^a^* Reference position is based on the USA300 FPR3757 genome sequence (NC_007793.1).

*^b^* Type of mutation with SNV indicating single nucleotide variant, DEL nucleotide deletion, and INS nucleotide insertions.

*^c^* Ref. indicates base in reference genome.

*^d^* Allele indicates base at the same position in sequenced strain.

*^e^* Freq. denotes frequency at which base changes were found in the sequence data.

*^f^* Average quality score.

*^g^* Where applicable, the resulting amino acid change in the protein found in the sequenced strain as compared to the reference strain; fs indicates frame shift and * indicates a stop codon.

##### An S. aureus Strain Producing a High Level of c-di-AMP Has Increased and a Stain Producing Low Levels Has Decreased Acid Resistance

**FIGURE 8.**
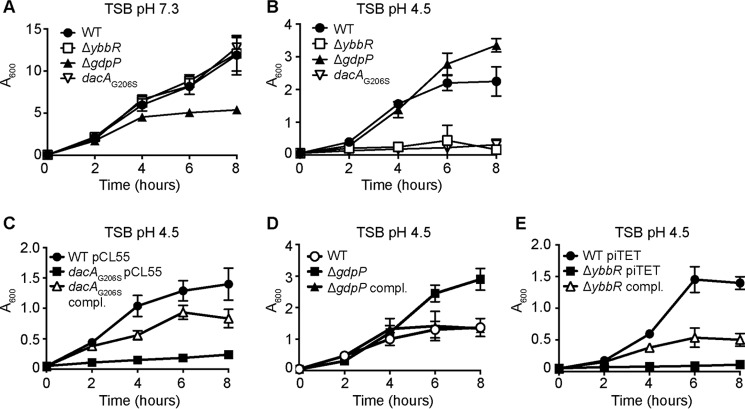
*S. aureus* strain LAC*Δ*ybbR* phenocopies the low c-di-AMP producing LAC*dacA_G206S_ strain when challenged with low pH. *A*, bacterial growth curves in TSB pH 7.3. Cultures of WT LAC* and the indicated mutant were diluted to an *A*_600_ of 0.05 and grown for 2.5 h. All cultures were then again back-diluted to an *A*_600_ of 0.05 (time = 0), and bacterial growth was monitored for 8 h by determining *A*_600_ readings at timed intervals. The average values and standard deviations from three independent experiments are plotted. *B–E*, bacterial growth curves in TSB, pH 4.5. Overnight cultures of WT LAC* and indicated mutant or complementation strains were back-diluted to an *A*_600_ of 0.05 in TSB pH 4.5 medium and grown for 3.5 h. All cultures were diluted again to an *A*_600_ of 0.05 (time = 0), and growth was monitored and plotted as described above.

It seemed likely that the DacA_G78V_ variant found in two of the suppressor strains has altered cyclase activity, leading to a change in the cellular c-di-AMP levels. Elevated levels of the dinucleotide have previously been linked to increased resistance to acid stress in *B. subtilis* and *Lactococcus lactis* (15, 38). To possibly unmask a correlation between c-di-AMP levels and acid resistance in *S. aureus,* WT LAC* and the high and low level c-di-AMP producing strains LAC*Δ*gdpP::kan* and LAC*dacA_G206S_ as well as strain LAC*Δ*ybbR* were cultured in TSB pH 7.3 or TSB pH 4.5 and growth was monitored over 8 h. Whereas LAC*, LAC*Δ*ybbR*, and LAC*dacA_G206S_ grew equally well in TSB pH 7.3 strain LAC*Δ*gdpP::kan* demonstrated the expected growth defect (Fig. 8*A*). When cultured in TSB, pH 4.5, however, strain LAC*Δ*gdpP::kan* grew better than WT, reaching a higher optical density at the 6- and 8-h time points (Fig. 8*B*). In contrast, the LAC*Δ*ybbR* and LAC*dacA_G206S_ strains struggled to grow in TSB pH 4.5 (Fig. 8*B*). Of note, LAC*dacA_G206S_ exhibited a clumping phenotype within 1.5 h under acid stress, LAC*Δ*ybbR* at 2 h, and WT at 4 h. No clumping was observed for strain LAC*Δ*gdpP::kan*. To confirm that the observed acid resistance and sensitivity phenotypes were due to a mutation in the respective genes, a complementation analysis was performed using strains LAC*dacA_G206S_ pCL55-*dacA* (Fig. 8*C*), LAC* *gdpP* compl. (Fig. 8*D*), and LAC*Δ*ybbR* piTET-*ybbR* (Fig. 8*E*) along with the appropriate control strains. Full complementation was observed for the *gdpP* complementation strain, whereas partial complementation was observed for strain LAC*dacA_G206S_ pCL55-*dacA* (Fig. 8*C*). The fact that the wild-type copy of *dacA* is expressed in addition to the mutated *dacA*_G206S_ gene could explain why only partial complementation was observed. Partial complementation was also observed for the *ybbR* complementation strain (Fig. 8*E*), but as shown earlier, reduced YbbR protein levels are produced by this strain as compared with WT LAC* (Fig. 6*C*). All together, these results suggest that high levels of c-di-AMP lead to increased acid tolerance, whereas low levels of c-di-AMP result in acid sensitivity.

##### c-di-AMP Levels Increase in WT as well as ybbR Mutant S. aureus Strains upon Exposure to Acid Stress

**FIGURE 9.**
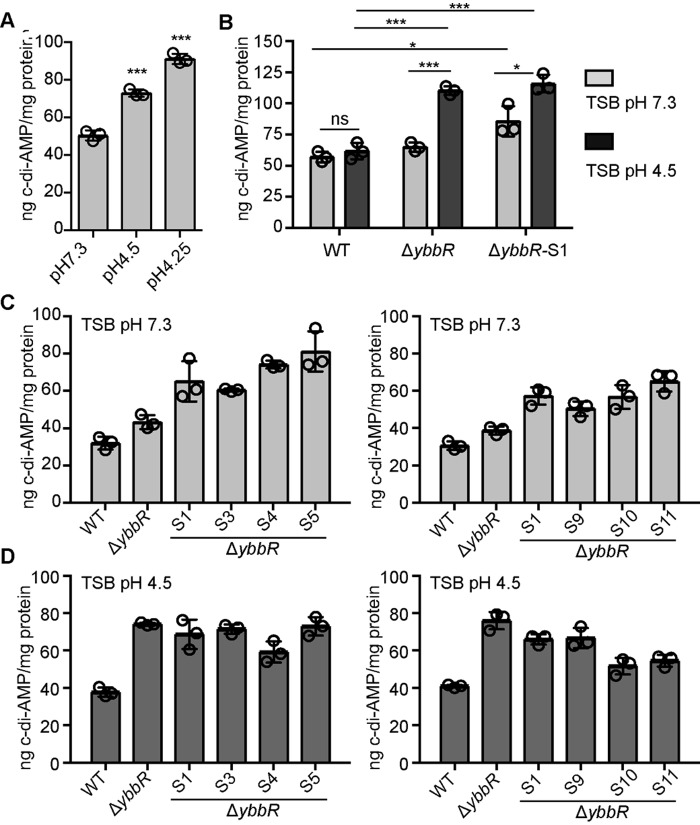
**c-di-AMP levels increase when *S. aureus* is exposed to low pH and are raised in the *ybbR* suppressor strains in standard growth medium.** *A–D,* determination of intracellular c-di-AMP levels by ELISA. *A,* cultures of WT LAC* were diluted to an *A*_600_ of 0.05 in TSB pH 7.3, pH 4.5, and pH 4.25. *B,* cultures of WT LAC* and the indicated mutant strains were diluted to an *A*_600_ of 0.05 in TSB pH 7.3 or pH 4.5. *C,* cultures of WT LAC* and the indicated mutant strains were diluted to an *A*_600_ of 0.05 in TSB pH 7.3 *D,* cultures of WT LAC* and the indicated mutant strains were diluted to an *A*_600_ of 0.05 in TSB pH 4.5. All cultures were grown to an *A*_600_ of 0.7, bacteria were harvested, and cell extracts were prepared. Lysates were normalized for protein concentration, cellular c-di-AMP levels were determined by ELISA, and values are reported as ng of c-di-AMP/mg protein. The average values and standard deviations from three independently prepared cell extracts are plotted. Statistical analysis was performed for *panels A* and *B* using an unpaired Student's *t* test where one (*) and three asterisks (***) indicate differences between WT and mutant strains or strains grown in TSB pH 7.3 *versus* low pH medium with a *p* value of <0.05 and <0.001, respectively. *ns*, not significant.

As described above, we observed a correlation between c-di-AMP levels and acid resistance in *S. aureus;* that is, a strain with increased levels of c-di-AMP is more resistant, whereas a low level strain has increased sensitivity to acid stress. Therefore, we set out to test if acid stress conditions would induce c-di-AMP production in WT *S. aureus*. This would point to the possible scenario where induction is blocked in the *ybbR* mutant strain, leading to increased acid sensitivity that can be rescued by the *ybbR* suppressor strain producing the DacA_G78V_ variant. The WT *S. aureus* strain LAC* was grown in TSB pH 7.3, TSB pH 4.5, and TSB pH 4.25, a pH at which the WT strain had a similar growth defect as the LAC*Δ*ybbR* when grown in TSB pH 4.5. Cytoplasmic extracts were then prepared, and c-di-AMP levels were measured by ELISA. Cellular c-di-AMP levels were indeed observed to increase when *S. aureus* was propagated in low pH medium (Fig. 9*A*). Next, we assessed c-di-AMP levels in the WT, the *ybbR* mutant, and the *ybbR*-S1 suppressor strain producing the DacA_G78V_ variant after growth in TSB pH 7.3 or TSB pH 4.5 medium. When grown in TSB pH 7.3 medium, the c-di-AMP levels in the suppressor strain *ybbR*-S1 were slightly but significantly increased as compared with the wild-type and *ybbR* mutant strains. Surprisingly, the c-di-AMP levels were found to increase in both the *ybbR* mutant and suppressor strain when grown in TSB pH 4.5 as compared with TSB pH 7.3 (Fig. 9*B*). These results indicate that the induction of c-di-AMP production is not blocked in the *ybbR* mutant strain. However, because a small but significant increase in the “basal” c-di-AMP level was noted for the LAC*Δ*ybbR*-S1 suppressor strain when grown in TSB pH 7.3 medium (Fig. 9*B*), this might suggest that the *dacA*_G206S_ variant has increased cyclase activity. Finally, to address if the increase in c-di-AMP production observed in the *ybbR* suppressor strain with a mutation in *dacA* is also observed in other suppressor strains, we determined the intracellular c-di-AMP levels of several other *ybbR* suppressor strains after their growth in TSB pH 7.3 or pH 4.5 medium. Interestingly the c-di-AMP levels in all suppressor strains were slightly increased as compared to the WT LAC* strain as well as the *ybbR* mutant strain in TSB pH 7.3 medium (Fig. 9*C*), and under acid stress conditions, the c-di-AMP levels remained slightly elevated in all suppressor strains (Fig. 9*D*). Taken together, these data highlight an important role for c-di-AMP in acid stress resistance in *S. aureus.*

## Discussion

**FIGURE 10.**
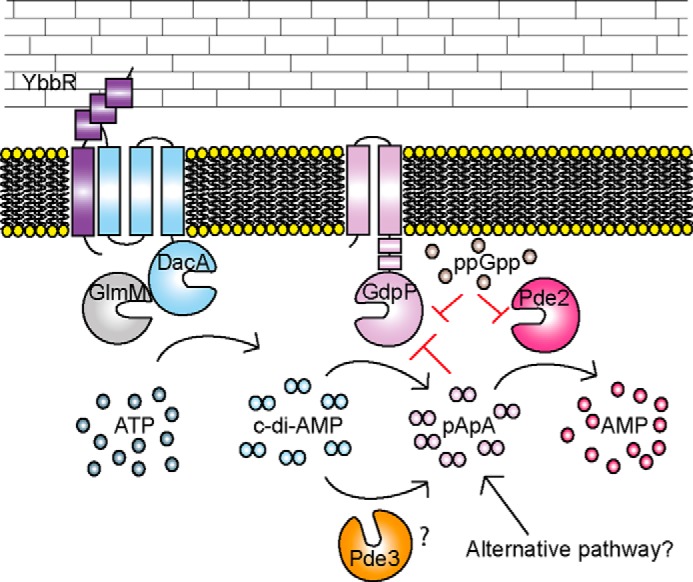
**Schematic representation of proteins involved in the c-di-AMP metabolism of *S. aureus*.** c-di-AMP is produced by the di-adenylate cyclase enzyme DacA, which is thought to form a complex with YbbR and GlmM. c-di-AMP is degraded by GdpP to pApA, which is further converted by the Pde2 enzyme to AMP. pApA can, in a feedback inhibition loop, reduce the activity of GdpP, and the activities of both GdpP and Pde2 are inhibited by the stringent response alarmone ppGpp. The results presented in this work suggest that yet another PDE Pde3, capable of degrading c-di-AMP to pApA, is present in *S. aureus* or that an alternative pathway for the production of pApA exists. The *S. aureus* DacA, GpdP, YbbR, and GlmM proteins have all been detected by Western blotting under standard laboratory growth conditions, and based on the observed phenotype of the *pde2* mutant strain it is expected that these proteins are produced and present at the same time.

The work performed in this study revealed a function of the second DHH/DHHA1 domain protein in *S. aureus,* which we have termed Pde2, as a c-di-AMP- and pApA-hydrolyzing PDE. Although Pde2 can degrade both c-di-AMP and pApA *in vitro*, biochemical assays and measurements of intracellular nucleotide levels indicated that pApA is the preferred substrate (Figs. 1–3). In the absence of Pde2, the intracellular pApA concentration increased drastically, and to the best of our knowledge, this is the first time that this dinucleotide has been detected in a bacterial cell (Fig. 3*C*). As part of this study, we also established that pApA can inhibit the c-di-AMP hydrolyzing activity of GdpP in a dose-dependent manner (Fig. 4, *A* and *B*, Fig. 10). Hence, Pde2 plays an important role in controlling intracellular pApA concentrations in *S. aureus* and prevents an increase in c-di-AMP levels either directly through its weak c-di-AMP hydrolase activity or indirectly through the pApA-dependent inhibition of GdpP. It is, however, likely that the enzyme has additional functions and nucleotide hydrolase activities in the cell that will be discussed.

Through recent studies it has become apparent that stand-alone DHH/DHHA1-domain enzymes can act on a variety of different nucleotide substrates ranging from nanoRNAs (RNA molecules ≤5 nucleotides), pApA, pGpG, cyclic-di-nucleotides, and pAp, which is a byproduct of sulfur assimilation and of acyl carrier protein production (39, 40). The *S. aureus* Pde2 enzyme shares a high degree of sequence similarity with the *B. subtilis* nanoRNase A NrnA (also referred to as YtqI), *M. tuberculosis* Rv2837c (also referred to as MtbPDE or CnpB), and *S. pneumoniae* Pde2 (also referred to as PapP). The latter two enzymes have been shown to degrade c-di-AMP and pApA to AMP, but all three enzymes also have confirmed nanoRNase and pAp-phosphatase activities (24, 29, 30, 32, 33, 39, 41). Based on these findings, it seems likely that *S. aureus* Pde2 may also degrade pAp and function as a general nanoRNA ribonuclease in the cell.

Although the *S. pneumoniae* and *S. aureus* Pde2 enzymes hydrolyze pApA more efficiently than c-di-AMP (Fig. 2 and Fig. 3, *A* and *B*) (24), this is not the case for all stand-alone DHH/DHHA1 enzymes involved in the metabolism of c-di-AMP characterized to date. Similar turnover rates toward c-di-AMP and pApA have been reported for the *M. tuberculosis* Rv2837c enzyme (32). In addition, this enzyme can degrade c-di-GMP and pGpG and was, therefore, not only implicated in c-di-AMP but also in c-di-GMP metabolism in *M. tuberculosis* (32). Detailed enzymatic *in vitro* studies combined with investigations into the effect that inactivation of these enzymes has on cellular nucleotide and ribonucleotide pools are, therefore, crucial in determining the actual physiological activities of DHH/DHHA1 domain enzymes.

The detection of pApA in *S. aureus* extracts derived from the *pde2* mutant strain (Fig. 3*C*) provides the first experimental support that this dinucleotide is a physiologically relevant substrate for this class of enzyme and present in the cell. Although we did not determine the cell size and exact μm amount of pApA in the *pde2* mutant strain, based on a comparison with the previously determined c-di-AMP amounts in a wild-type *S. aureus* strain grown under similar conditions, we estimate that pApA reaches a cellular concentration of ∼25 μm in the *pde2* mutant. It is possible that under certain conditions when Pde2 function is inhibited, pApA levels may also be high in a wild-type strain. This is an interesting possibility, as it has previously been speculated that the c-di-AMP breakdown product pApA could potentially act as a nanoRNA source in bacteria (42), an increase that can result in changes in gene expression through differential priming of transcription (43). Surprisingly, an accumulation of pApA was also observed in a *S. aureus* mutant strain lacking both GdpP and Pde2 (Fig. 3*C*), highlighting that besides these two enzymes, another c-di-AMP PDE might be present (Fig. 10). As GdpP and Pde2 are the only DHH/DHHA1 domain proteins annotated in the *S. aureus* proteome, an enzyme with another domain architecture must be responsible for this activity. Alternatively, pApA could be derived as a byproduct from a different cellular pathway (Fig. 10), an interesting possibility that will be investigated in future studies.

We show here that pApA can inhibit the activity of the c-di-AMP PDE GdpP (Fig. 4, *A* and *B*). This might be an alternative explanation as to why, upon deletion of *pde2*, an increase in the concentration of c-di-AMP is evident in *S. aureus* (Fig. 3*C*). The regulation of GdpP is even more complex, as its activity is also inhibited, at least *in vitro*, by the stringent response nucleotide ppGpp (15, 35). Interdependencies between cellular (p)ppGpp and c-di-AMP levels have been reported in several studies (35, 44). Although the molecular mechanisms behind this have not yet been well studied, one direct link between these two signaling molecules appears to be the c-di-AMP hydrolyzing enzymes such as GdpP and, as shown in this study, the pApA-degrading enzyme Pde2 (Fig. 4, *C* and *D*, and Fig. 10). The link between c-di-AMP and (p)ppGpp-controlled pathways is, however, a lot more complex and is not solely controlled by the inhibition of c-di-AMP- and pApA-metabolizing enzymes by (p)ppGpp as it has also been shown that the absence of c-di-AMP leads to a toxic increase in (p)ppGpp levels (44).

Our study has also provided further insight into the cellular function of YbbR (also referred to as CdaR). YbbR homologs have been shown to directly interact with DacA homologs (also referred to as CdaA) in other bacteria (21–23). In addition, an interaction between CdaA and GlmM has been reported, leading to the hypothesis that DacA, YbbR, and GlmM form a three-protein complex (22, 32) (Fig. 10). We have identified a new role for YbbR in acid-stress resistance in *S. aureus* and also provide further information on how c-di-AMP levels impact acid tolerance in this organism (Figs. 6 and 9).

In this study we show that a *S. aureus gdpP* mutant strain that produces high levels of c-di-AMP displays enhanced acid resistance (Fig. 8, *B* and *D*), as has been reported for *L. lactis, L. monocytogenes,* and *B. subtilis gdpP* mutant strains (15, 25, 38). Expanding on this, we further show that a *dacA*_G206S_ mutant *S. aureus* strain, which produces low levels of c-di-AMP, demonstrates decreased acid resistance (Fig. 8, *B* and *C*). Furthermore, c-di-AMP levels increased in a wild-type *S. aureus* strain when grown in acidified medium (Fig. 9*A*), a response that likely helps this organism cope with this stress condition. *S. aureus* can colonize a variety of sites within the human host that vary in pH between 4.2 and 7.6 (45). It is, therefore, vital that the bacterium mounts a suitable response to survive these challenging conditions, and this work highlights that c-di-AMP likely plays an important role in this. There are a number of characterized mechanisms that bacteria utilize in an effort to survive acidic environments. These include expelling cytoplasmic protons through the F_1_F_0_-ATPase, producing alkaline compounds to neutralize the cytoplasmic pH, and importing osmolytes such as choline or proline (46, 47). Microarray data from *S. aureus* cultured in medium acidified to pH 4.5 revealed that besides genes encoding urease and proton efflux pumps, oxidative stress response genes and the *kdp* and *opuC* operons, encoding the c-di-AMP receptor proteins KdpD and OpuCA, are up-regulated (45, 48–51). Although an intriguing correlation, understanding the mechanism through which elevated c-di-AMP levels contributes to the acid tolerance in *S. aureus* or other microorganisms awaits future studies.

The *ybbR* mutant strain exhibited decreased acid resistance (Fig. 8), and therefore, we expected this strain to produce low concentrations of c-di-AMP under acid stress conditions. Surprisingly, cellular c-di-AMP concentrations were increased in the *ybbR* mutant strain as compared with wild-type when grown in low pH medium (Fig. 9*B*). Whole genome sequencing of LAC*Δ*ybbR* suppressor strains that had acquired the ability to grow on acidified medium offered some insight into the mechanisms used by these strains to survive in the absence of YbbR. All of these suppressor mutations caused a slight increase in the basal c-di-AMP levels in the cell when the bacteria were grown under our standard growth conditions in TSB pH 7.3 medium (Fig. 9*C*), and we believe that this increase in c-di-AMP levels might help these bacteria to cope with the acid stress. For a number of the suppressor strains it is harder to speculate on the connection between their genomic alterations, the increase in c-di-AMP production under standard laboratory conditions, and how the mutation leads to a reversion in acid sensitivity. However, it is interesting to note that a premature stop codon was identified in *escA*, a gene encoding an ABC transporter ATP-binding protein, and a frameshift mutation in the predicted membrane-associated zinc metalloprotease RseP (Table 1). *B. subtilis* orthologs of these two proteins have been shown to function in the same cellular pathway, although a connection with the acid stress response is at this point not clear. It has been shown that the ABC transporter EcsAB is required for the activity of the intramembrane cleaving protease RasP (the RseP ortholog in *B. subtilis*), and *escA* and *rasP* mutants show similar signal sequence processing and protein secretion defects (52, 53). Two independent mutations in an annotated proline/glycine betaine transporter were identified, one of which resulted in a premature stop codon. Osmolyte uptake is known to be important in maintaining pH homeostasis and for bacterial osmoprotection (47, 54). It is possible that deactivation or decreased proline/glycine betaine transporter activity allows for the accumulation of a more efficient compatible solute. In fact, deletion of a putative glycine betaine transporter from *Salmonella enterica* serovar Typhimurium led to increased resistance to salt stress and low pH as long as trehalose was produced (55). Perhaps two of the most interesting LAC*Δ*ybbR* suppressor strains obtained in two independent screens contained the same valine-to-glycine substitution at amino acid position 79 in DacA. It is possible that this substitution may result in a change in the conformation of DacA, affect DacA dimerization, its interaction with GlmM, or its ability to sense stimuli. Based on cellular c-di-AMP measurements, the ultimate consequence of this appears to be an increase in cyclase activity under “non-stressed” standard growth conditions (Fig. 9*B*). This increase in c-di-AMP activity may now allow a *ybbR* mutant strain to survive under acid stress conditions. Altogether, this work provides not only new insights into the c-di-AMP metabolism of *S. aureus* but also opens up a number of exciting possibilities for future investigations.

## Experimental Procedures

### 

#### 

##### Bacterial Strains and Culture Conditions

*E. coli* strains were cultured in Luria Bertani (LB) medium and *S. aureus* strains in TSB at 37 °C with aeration unless otherwise stated. All bacterial strains used in this study are listed in Table 2 along with the relevant antibiotics and inducers used for their propagation. For growth under acidic conditions, the pH of TSB, which usually has a pH of 7.3, was lowered to pH 4.5 or pH 4.25 with HCl, and for growth on plates, 30 g of agar per liter TSB was added before lowering the pH.

**TABLE 2:** **Bacterial strains used in this study** Antibiotics were used at the following concentrations: for *E. coli* cultures: 100 μg/ml ampicillin (AmpR), 10 μg/ml chloramphenicol (CamR), and 30 μg/ml kanamycin (KanR); for *S. aureus* cultures: chloramphenicol (CamR), 10 μg/ml for plasmids and 7.5 μg/ml for integrated plasmids; 10 μg/ml erythromycin (ErmR), and 90 μg/ml kanamycin (KanR). The inducer anhydrotetracycline (Atet) was used at a concentration of 200 ng/ml in agar and 50 ng/ml in broth.

Strain name	Description	Reference
***Escherichia coli***		
XL1-Blue	Cloning strain, TetR, ANG127	Stratagene
BL21(DE3)	Protein expression strain, ANG191	Novagen
DC10B	Cloning strain, *dcm*, ANG2151	(58)
DH10B pIMAY	DH10B pIMAY; CamR, ANG2154	(58)
IM08B	*E. coli* strain expressing a *S. aureus* DNA modification system; ANG3724	(62)
ANG243	XL1-Blue pCL55; *S. aureus* single-site integration vector: AmpR	(63)
ANG284	XL1-Blue piTET; AmpR	(64)
ANG1824	XL1-Blue pET28b; KanR	Novagen
ANG1861	BL21 (DE3) pET28b-*gdpP*_84–655_; KanR	(7)
ANG2262	XL1-Blue pET28b-*ybbR*_noTM_; KanR	This study
ANG2281	BL21 (DE3) pET28b-*ybbR*_noTM_; KanR	This study
ANG3048	BL21 (DE3) pET28b-*disA*_BT_-His*;* KanR	(65)
ANG3472	XL1-Blue piTET-*ybbR*; AmpR	This study
ANG3480	XL1-Blue pET28b-His-*cabP*_SP_; KanR	This study
ANG3481	BL21 (DE3) pET28b-His-*cabP*_SP_; KanR	This study
ANG3511	H10B pIMAYΔ*pde2*::*erm*; CamR	This study
ANG3544	XL1-Blue pIMAYΔ*pde2*::*erm*; CamR	This study
ANG3732	IM08B pCL55; AmpR	(51)
ANG3898	XL1-Blue pET28b-His-*pde2*; KanR	This study
ANG3899	BL21 (DE3) pET28b-His-*pde2*; KanR	This study
ANG3606	XL1-Blue pIMAY-*dacA*_G206A_; CamR	This study
ANG3962	XL1-Blue pIMAY-*gdpP*; CamR	This study
ANG3963	IM08B pIMAY-*gdpP*; CamR	This study
ANG3990	XL1-Blue pIMAYΔ*ybbR*; CamR	This study
ANG3991	DC10B pIMAYΔ*ybbR*; CamR	This study
ANG4123	XL1-Blue pCL55-*pde2*; AmpR	This study
ANG4124	XL1-Blue pCL55-*dacA*; AmpR	This study
ANG4125	IM08B pCL55-*pde2*; AmpR	This study
ANG4126	IM08B pCL55-*dacA*; AmpR	This study

***Staphylococcus aureus***		
RN4220	Restriction-deficient derivative of 8325-4	(66)
LAC*	CA-MRSA LAC strain; ErmS; ANG 1575	(67)
ANG290	RN4220 piTET	This study
ANG1961	LAC*Δ*gdpP*::*kan*; KanR	(7)
ANG3301	LAC*Δ*ybbR*	This study
ANG3516	RN4220 pIMAYΔ*pde2*::*erm*; CamR ErmR 28 °C	This study
ANG3547	RN4220 piTET-*ybbR*; CamR	This study
ANG3548	LAC*Δ*ybbR* piTET-*ybbR*; CamR	This study
ANG3559	LAC* Δ*pde2*::*erm*; ErmR	This study
ANG3652	RN4220 pIMAY-*dacA_G206S_*; CamR 28 °C	This study
ANG3659	LAC* pIMAY-*dacA_G206S_*; CamR 28 °C	This study
ANG3664	LAC*dacA_G206S_	This study
ANG3726	LAC*Δ*gdpP*::*kan* Δ*pde2*::*erm*; KanR ErmR	This study
ANG3817	LAC*Δ*ybbR*-S1 (suppressor strain 1 )	This study
ANG3818	LAC*Δ*ybbR-*S2 (suppressor strain 2)	This study
ANG3819	LAC*Δ*ybbR-*S4 (suppressor strain 4)	This study
ANG3820	LAC*Δ*ybbR-*S5 (suppressor strain 5)	This study
ANG3821	LAC*Δ*ybbR-*S7 (suppressor strain 7)	This study
ANG3822	LAC*Δ*ybbR-*S8 (suppressor strain 8)	This study
ANG3823	LAC*Δ*ybbR-*S9 (suppressor strain 9)	This study
ANG3824	LAC*Δ*ybbR-*S10 (suppressor strain 10)	This study
ANG3825	LAC*Δ*ybbR-*S11 (suppressor strain 11)	This study
ANG3855	LAC*Δ*ybbR-*S3 (suppressor strain 3)	This study
ANG3869	LAC*Δ*ybbR* piTET; CamR	This study
ANG3795	LAC* pCL55	(51)
ANG3988	LAC* piTET	This study
ANG4003	RN4220 pIMAYΔ*ybbR*; CamR, 28 °C	This study
ANG4040	LAC*gdpP compl.	This study
ANG4045	LAC* pIMAYΔ*ybbR*; CamR, 28 °C	This study
ANG4127	LAC*Δ*pde2* pCL55; CamR	This study
ANG4128	LAC*Δ*pde2* pCL55-*pde2*; CamR	This study
ANG4129	LAC*dacA_G__206S_ pCL55; CamR	This study
ANG4130	LAC*dacA_G206S_ pCL55-*dacA*; CamR	This study

##### Strain Construction

*E. coli* and *S. aureus* strains used in this study are listed in Table 2, and the primers used are in Table 3. Plasmid pET28b-His-*pde2* was constructed for the expression and purification of the N-terminally His-tagged *S. aureus* Pde2 protein. To this end, the *pde2* gene was amplified by PCR from LAC* chromosomal DNA using primers ANG2214 and ANG2215. The product was digested with NheI and EcoRI and ligated with the similarly digested vector pET28b. Plasmid pET28b-His-*pde2* was initially recovered in *E. coli* XL1-Blue and then introduced into *E. coli* BL21 (DE3) for protein production, yielding strains ANG3898 and ANG3899, respectively. Plasmid pET28-His-*cabP*_SP_ was constructed for the expression and purification of the His-tagged *S. pneumoniae* c-di-AMP receptor protein CabP (56). To this end, the *cabP* gene was amplified from *S. pneumoniae* strain D39 chromosomal DNA using primers ANG2034 and ANG2035. The resulting PCR product was digested with NdeI and HindIII and ligated with the similarly digested pET28b. The plasmid was then transformed into *E. coli* XL1-Blue yielding strain ANG3480 and subsequently introduced into *E. coli* strain BL21(DE3) for protein expression, giving strain ANG3481. Plasmid pET28b-*ybbR*_noTM_was generated for the expression and purification of the soluble YbbR protein domains, a protein that was subsequently used for antibody production. The *ybbR* gene (minus the predicted transmembrane region) was amplified from LAC* DNA using primers ANG1370 and ANG1371 and digested with BamHI and NcoI. The product was ligated into similarly digested pET28b and transformed into XL1-Blue yielding strain ANG2262 and subsequently into BL21(DE3) yielding strain ANG2281.

**TABLE 3:** **Primers used in this study** Restriction site sequences are underlined.

Primer	Name	Sequence
ANG1370	R_YbbR_noTM_	CGGGATCCCGTTTTACATTTATATAAGCCTTCGTTTCAC
ANG 1371	F_YbbR_noTM_	CATGCCATGGCTGGTAATCTTGGTCAAAAGTCTAGTAAAAC
ANG1765	F_EcoRI_dacA	GCGCGAATTCTATAAAGTATATTTTGCTTTTTGC
ANG1848	F_KpnI_YbbRdel1	GCGCGGTACCAATTGTAACGAGTATCCTTG
ANG1814	R_YbbRdel1	GCCTTCGTTTCAAATCTCAAGCCCCATTTACTTTC
ANG1815	F_YbbRdel2	GGCTTGAGATTTGAAACGAAGGCTTATATAAATGTA
ANG1816	R_EcoRI_YbbRdel2	GCGCGAATTCAACGATATGTCCAGATTGTTCTCC
ANG1817	F_YbbRdel_check	GCAATGATTATTCAAGGCACGAAG
ANG1818	R_YbbRdel_check	CCCATATCGCGTGTTAAATATGC
ANG1908	F_AvrII_YbbR	GCGCCCTAGGCATTGGTTTGGCACACGCTTTC
ANG1909	R_SacII_YbbR	GCGCCCGCGGTTATTTTACATTTATATAAGCCTTCG
ANG2034	F_NdeI_CabP_Nhis	TTTCATATGTCAGATCGTACGATTGGAATTTTGGGC
ANG2035	R_HindIII_CabP	TTTAAGCTTACGAATTCAATGCTACTAGGGTATCC
ANG2040	F_EcoRI_1650del1	GCGCGAATTCGTTCAAGATATAATGACGCCATTAG
ANG2041	R_1650del1	GAAAAAGGAAGAGTCAAAGTACTAATCATTTTCATACTCCC
ANG2042	F_1650del2	GATTAGTACTTTGACTCTTCCTTTTTCAATATTATTGAAGC
ANG2043	R_1650del2	GTTAAGTTTTGTTTATTTCCTCCCGTTAAATAATAGATAAC
ANG2044	F_1650del3	CGGGAGGAAATAAACAAAACTTAACTAATAGAAAGGGGCC
ANG2045	F_KpnI_1650del3	GCGCGGTACCCCAATAAATAACTGACCAGTGAGCC
ANG2086	F_1650del_check	CGGAAATCGTGAAGATGTGCAGATTG
ANG2087	R_1650del_check	CCAACATCTCTAGCAACTGCTCTTGC
ANG2104	F_EcoRI_dacA_G206A_1	CGGAATTCCCAGGCACTGGTACTAGAGTTTTGGGTGGTC
ANG2106	R_XmaI_dacA_G206A_2	TCCCCCCGGGCATTTATATAAGCCTTCGTTTCACTTGGTTG
ANG2109	R_dacA_G206A_1	GCTCTATGTCTTGTcgaCAAACTTTTAGATATCTTAGGACTATCAGAC
ANG2110	F_dacA_G206A_2	CTAAAAGTTTGtcgACAAGACATAGAGCTGCGGTTGGTATTTCAG
ANG2214	F_NheI_Pde2_Nhis	GCGCGCTAGCATGATTAGTACTTTGAATGAAATTATG
ANG2215	R_EcoRI_Pde2	GCGCGAATTCTTAGTTAAGTTTTGTGCGTAAAGCTGTAGC
ANG2254	F_KpnI_gdpP	GACTCGGTACCTTCAATTAAATGAAATAGAAGCATACAATC
ANG2255	R_NotI_gdpP	GAATAGCGGCCGCCTCTTCAGCTGTTTCATACACTTGTC
ANG2472	F_EcoRI_1650	GCGCGAATTCGAACTTAGCAGAGAAAATAGGCATAAG
ANG2473	R_BamHI_1650	GCGCGGATCCTTAGTTAAGTTTTGTGCGTAAAGCTG
ANG2474	R_BamHI_dacA	GCGCGGATCCTTATTTCACACCTTTCTTTTGAAAGCGTG

To generate the *erm*-marked *pde2* mutant strain LAC*Δ*pde2::erm* (ANG 3559), 1-kb DNA fragments up- and downstream of the *pde2* gene were amplified by PCR from LAC* genomic DNA using primer pairs ANG2040/2041 and ANG2044/2045. The erythromycin antibiotic resistance gene *ermC* was amplified from plasmid pCN49 (57) using primers ANG2042 and ANG2043. The upstream DNA and *ermC* fragments were fused by splice overlap extension (SOE) PCR using primers ANG2040 and ANG2043, and the resulting PCR product was then fused to the downstream DNA fragment using primers ANG2040 and ANG2045. This PCR product was digested with EcoRI and KpnI and cloned into pIMAY digested with the same restriction enzymes, yielding plasmid pIMAYΔ*pde2::erm*. The plasmid was initially recovered in *E. coli* strain XL-1 Blue and subsequently introduced into the *dcm* mutant *E. coli* strain DC10B, yielding strains ANG3544 and ANG3511. The pIMAYΔ*pde2::erm* construct was then electroporated into *S. aureus* RN4220 yielding strain ANG3516 and subsequently into *S. aureus* LAC*, which was propagated at 28 °C. Allelic exchange was performed as described by Monk *et al.* (58), finally yielding strain LAC*Δ*pde2::erm* (ANG3559). The*gdpP/pde2* double mutant strain LAC*Δ*gdpP*::*kan*Δ*pde2*::*erm* (ANG3726) was generated by transducing the *gdpP*::*kan* mutation from strain ANG1961 into strain ANG3359 using phage Φ85, and successful transduction was confirmed by PCR using primers ANG2086 and ANG2087. The complementation strain LAC* Δ*pde2* pCL55-*pde2* (ANG4128) was generated by first amplifying the *pde2* gene (including its promoter) from LAC* genomic DNA using primers ANG2472 and ANG2473. The PCR product was digested with EcoRI and BamHI and ligated with the similarly digested plasmid pCL55. The resulting plasmid pCL55-*pde2* was recovered in *E. coli* XL1-Blue and subsequently introduced into *E. coli* strain IM08B, yielding strains ANG4123 and ANG4125, respectively. The plasmid was isolated from strain ANG4125 and introduced and integrated into the *geh* locus of *S. aureus* LAC*Δ*pde2*::*erm* (ANG3559), yielding strain LAC*Δ*pde2*::*erm* pCL55-*pde2* (ANG4128). As control strains, the empty pCL55 vector was isolated from strain IM08B pCL55 (ANG3732) (51) and introduced into strains LAC* and LAC*Δ*pde2*::*erm*, yielding strains LAC* pCL55 (ANG3795) (51) and LAC*Δ*pde2*::*erm* pCL55 (ANG4127), respectively. The *gdpP* complementation strain LAC*gdpP compl. was generated by replacing the kanamycin-marked deletion of LAC*Δ*gdpP::kan* (ANG1961) with a complete version of *gdpP* gene. First, the entire *gdpP* gene including 1-kb up- and downstream fragments was amplified from LAC* genomic DNA using primers ANG2254 and ANG2255. The resulting PCR product was digested with KpnI and NotI, inserted into the similarly digested pIMAY plasmid, and recovered in *E. coli* XL-Blue to yield strain ANG3962. The plasmid was then transformed into *E. coli* IM08B and subsequently introduced into LAC*Δ*gdpP::kan* (ANG1961). Allelic exchange was performed as described by Monk *et al.* (58) yielding strain LAC*gdpP compl. (ANG4040). Repair of the *gdpP* locus was confirmed by PCR and sequencing. To generate the *S. aureus ybbR* deletion strain LAC*Δ*ybbR* (ANG3301), 1-kb DNA fragments up- and downstream of *ybbR* were amplified from LAC* genomic DNA using primer pairs ANG1848/ANG1814 and ANG1815/ANG1816. The PCR products were fused by SOE PCR using primers ANG1848 and ANG1816, digested with EcoRI and KpnI, and ligated with similarly digested pIMAY, yielding plasmid pIMAYΔ*ybbR*. The plasmid was recovered in *E. coli* XL1-Blue (giving strain ANG3990), shuttled through DC10B (strain ANG3991), and subsequently electroporated into RN4220 and LAC*, yielding strains ANG4003 and ANG4045, respectively. The allelic exchange was performed as described above, yielding strain LAC*Δ*ybbR* (ANG3301), and the *ybbR* deletion was confirmed by PCR using primers ANG1817 and ANG1818. The plasmid piTET-*ybbR* for Atet-inducible expression of *ybbR* in *S. aureus* was generated by amplifying the *ybbR* gene from LAC* chromosomal DNA using primers ANG1908 and ANG1909. The PCR product and plasmid piTET were digested with AvrII and SacII, ligated, and then transformed into *E. coli* XL1-Blue yielding strain XL1-Blue piTET-*ybbR* (ANG3472). The plasmid was subsequently introduced and integrated into the *geh* locus of *S. aureus* strain RN4220, yielding strain RN4220 piTET-*ybbR* (ANG3547), and subsequently moved by phage transduction using phage Φ85 into strain LAC*Δ*ybbR* to generate the *ybbR* complementation strain LAC*Δ*ybbR* piTET-*ybbR* (ANG3548). As controls, strains LAC* piTET (ANG3988) and LAC*Δ*ybbR* piTET (ANG3869), containing the empty piTET vector were produced. To this end, plasmid piTET was first integrated into the *geh* locus of *S. aureus* strain RN4220, yielding strain RN4220 piTET (ANG290), and subsequently moved by phage transduction using phage Φ85 into strain LAC* and LAC*Δ*ybbR*, respectively.

An *S. aureus* strain producing a DacA variant in which the glycine at amino acid position 206 is replaced with a serine (from here on out referred to as *dacA*_G206S_ allele) has been shown to produce reduced levels of c-di-AMP (26). To generate the low c-di-AMP level *S. aureus* strain LAC*dacA_G206S_, primer pairs ANG2104/ANG2109 and ANG2106/2110 were used to amplify 1-kb fragments upstream and downstream from the *dacA* Gly-206 codon using LAC* genomic DNA in PCRs, and the resulting products were fused by SOE PCR using primers ANG2104 and ANG2110. Primer ANG2109 and ANG2110 were designed to introduce the Gly-206-to-Ser substitution. The resulting PCR product was then digested with EcoRI and XmaI and ligated into pIMAY digested with the same enzymes. Plasmid pIMAY*dacA*_G206S_ was initially recovered into *E. coli* strain XL1-Blue to yield ANG3606, and the plasmid was shuttled through *S. aureus* RN4220 to generate strain RN4220 pIMAY-*dacA*_G206S_ (ANG3652) and subsequently introduced into strain LAC* to produce LAC* pIMAY-*dacA*_G206S_ (ANG3659) that was propagated at 28 °C. The allelic exchange was performed as described above yielding strain LAC*dacA_G206S_ (ANG3664). The substitution of the glycine to serine codon in *dacA* as well as all other plasmid inserts was verified by fluorescence-automated sequencing. To generate the complementation strain LAC*dacA_G206S_ pCL55-*dacA*, the *dacA* gene (including its promoter) was amplified from LAC* genomic DNA using primers ANG1765 and ANG2474. The product was digested with EcoRI and BamHI and inserted into the similarly digested plasmid pCL55. Plasmid pCL55-*dacA* was recovered in *E. coli* XL1-Blue and subsequently introduced into *E. coli* IM08B, resulting in the strains ANG4124 and ANG4126, respectively. The plasmid was then electroporated into strain LAC*dacA_G206S_ (ANG3664) yielding strain LAC*dacA_G206S_ pCL55-*dacA* (ANG4130).

##### Growth Curves

*S. aureus* strains were grown overnight in TSB medium containing the relevant antibiotic. Overnight cultures of WT LAC* and the indicated mutant strains were diluted in fresh TSB medium to an *A*_600_ of 0.01 or diluted to an *A*_600_ of 0.05 if strain LAC*Δ*gdpP*::*kan* was included in the growth curve. Cultures were incubated at 37 °C with aeration until an *A*_600_ of 1 was reached. Fresh TSB was inoculated with the starter culture to an *A*_600_ of 0.01 or 0.05 as before, and growth was monitored for 8 h by taking *A*_600_ readings every 2 h. Growth curves in TSB pH 4.5 medium were performed as follows. *S. aureus* strains were grown overnight in TSB medium before diluting in TSB pH 4.5 to an *A*_600_ of 0.05. Cultures were incubated at 37 °C with aeration for 3.5 h before diluting again into fresh TSB pH 4.5 medium to an *A*_600_ of 0.05. Growth was then monitored for 8 h by taking *A*_600_ readings every 2 h. Alongside this, the same strains were cultured in TSB medium and treated similarly as a control. The first dilution was made into TSB medium, but cultures were grown for 2.5 h before diluting into fresh TSB medium once more. For the *pde2* growth complementation assay in TSB 7.3 medium, overnight cultures of the indicated strains were diluted to an *A*_600_ of 0.01, and growth was monitored for 10 h by taking *A*_600_ readings every 2 h. All growth curves were performed in triplicate with average values and standard deviations plotted.

##### Oxacillin Minimum Inhibitory Concentrations

Overnight cultures of WT LAC* and LAC* mutant strains were diluted to an *A*_600_ of 0.1 before spreading on Mueller-Hinton agar plates containing 2% NaCl. Oxacillin M.I.C. Evaluator^TM^ (ThermoScientific) strips were then applied to the agar, and the plates were incubated at 35 °C for 24 h. MIC values represent an average of three independent experiments.

##### Protein Purifications

His-tagged *S. aureus* GdpP, Pde2, and YbbR_noTM_ proteins and the His-tagged *Bacillus thuringiensis* DisA protein (DisA_BT_) were purified from 1 or 2 liters of *E. coli* BL21(DE3) cultures. After overnight growth at 30 °C, strains were diluted 1:20 and cultured to an *A*_600_ of 0.5–0.8. Protein expression was then induced with 0.5 mm isopropyl 1-thio-β-d-galactopyranoside. Strains were incubated overnight at 16 °C for GdpP and YbbR_noTM_ overexpression at 37 °C for 4 h in the case of Pde2 and overnight at 18 °C for DisA_BT_. Proteins were purified by nickel affinity and size exclusion chromatography as previously described (7). His-tagged CabP from *S. pneumonia* was expressed and purified essentially as described by Bai *et al.* (56). Briefly, 1 liter of BL21(DE3) pET28b-His-*cabP*_SP_ culture was grown at 37 °C to an *A*_600_ of 0.6, and protein expression was induced with 0.1 mm isopropyl 1-thio-β-d-galactopyranoside for 4 h at 24 °C. Bacteria were collected by centrifugation and suspended in 20 ml of 50 mm Tris, pH 7.5, 500 mm NaCl, 10% glycerol lysis buffer containing 10 mm imidazole, and cells were lysed by French pressing. The His-CabP_SP_ protein was purified by nickel affinity chromatography, where the column was washed after protein loading with 10 column volumes lysis buffer containing 10 mm, 20 mm, and finally 30 mm imidazole and subsequently eluted in 4 ml of lysis buffer containing 250 mm imidazole. The protein was subsequently dialyzed at 4 °C twice for 2 h against 1 liter of phosphate-buffered saline (PBS), pH 7.4, buffer and finally overnight against 1 liter of PBS, pH 7.4, buffer containing 10% glycerol. Protein concentrations were determined using the Pierce BCA kit, snap-frozen, and stored at −80 °C.

##### Western Blotting

Bacteria from 0.5–1-ml cultures of the different *S. aureus* strains were harvested by centrifugation and suspended in TM buffer (50 mm Tris-HCl, pH 7.5, 10 mm MgCl_2_) containing 100 μg/ml lysostaphin and 20 μg/ml DNase. Samples were normalized for *A*_600_ readings of the original cultures; that is, cells collected from a 1-ml culture with an *A*_600_ of 1 were suspended in 15 μl of TM buffer. Samples were incubated at 37 °C for 30 min and subsequently mixed with an equal volume of 2× SDS protein sample buffer. This was followed by boiling the samples for 10 min and centrifugation for 5 min. Samples were then separated on 12% SDS-polyacrylamide gels and transferred to PVDF membranes. DacA and YbbR were detected using rabbit polyclonal anti-DacA (35) or anti-YbbR antibodies (produced at Covalab as part of this study) at 1:10,000 and 1:20,000 dilutions, respectively, and SrtA was detected using a rabbit polyclonal anti-SrtA antibody (59) at a dilution of 1:20,000. An HRP-conjugated anti-rabbit IgG secondary antibody (Cell Signaling Technologies, catalogue no. 7074) was used at a 1:10,000 dilution. Blocking and antibody incubation steps were performed in the presence of 10 μg/ml human IgG to reduce unspecific antibody binding to *S. aureus* Spa and Spi proteins. Blots were developed using Clarity^TM^ Western ECL Blotting Substrate (Bio-Rad) and imaged using the ChemiDoc^TM^ Touch Imaging System (Bio-Rad). Western blottings were performed in triplicate, and a representative result is shown.

##### Quantification of c-di-AMP and pApA by LC-MS/MS

Starter cultures of the WT LAC* *S. aureus* strain and the different mutant strains were prepared in 5 ml of TSB medium and grown for 4 h at 37 °C with aeration. The cultures were back-diluted to an *A*_600_ of 0.05 in 5 ml if TSB medium and grown overnight in the same conditions. Cells were harvested from the overnight cultures, and bacterial cell extracts were prepared and analyzed as described previously using 0.58 μm ^13^C,^15^N isotope-labeled c-di-AMP as an internal standard (7). At the same time the dry weight of the bacteria from a culture aliquot was determined for normalization purposes. For pApA measurements, 0.58 μm ^13^C,^15^N isotope-labeled c-di-AMP was added to the samples after extraction and before the LC-MS/MS analysis to account for internal ion suppression. c-di-AMP and pApA concentrations within the samples were determined based on a standard curve produced with known concentrations of c-di-AMP and pApA, respectively. Values are reported as ng of c-di-AMP/mg cell weight or ng of pApA/mg cell weight, and the averages values and standard deviations from three samples were plotted. A two-tailed two-sample equal variance Student's *t* test was used to determine statistically significant differences between c-di-AMP and pApA levels in WT *versus* mutant strains.

##### Detection and Quantification of Nucleotides by LC-UV

100 μm unlabeled c-di-AMP or pApA (BioLog) were mixed with 1 μm purified Pde2 enzyme in 50 mm Tris-HCl, pH 7.5, 0.1 mm MnCl_2_, and 20 mm KCl buffer and incubated at 37 °C for 2 h. Reactions were stopped by boiling for 10 min, and precipitated proteins were subsequently removed by centrifugation. The LC-UV analysis of the reaction products was performed as previously described (60) with the following modifications. Instead of mass spectrometric analysis, a Dionex HPLC-UV system with a constant flow rate of 0.6 ml/min and a wavelength at 259 nm was applied. Solutions with known concentrations of AMP (retention time 3.0 min), c-di-AMP (retention time 8.6 min), and pApA (retention time 9.0 min) were used as calibrators.

##### Synthesis of Radiolabeled c-di-AMP and pApA

c-di-AMP used in *in vitro* nucleotide conversion assays was synthesized by incubating 2-mm unlabeled ATP spiked with 9.99 nm ^32^P-labeled ATP (PerkinElmer Life Sciences) with 10 μm DisA_BT_ in 40 mm Tris-HCl, pH 7.5, 100 mm NaCl, 10 mm MgCl_2_ buffer. This reaction was incubated at 45 °C for 24 h and deactivated by heating to 95 °C for 10 min, and the protein was removed by centrifugation at 13,000 rpm for 5 min. 1 mm c-di-AMP was converted to pApA using 3 μm GdpP in 50 mm Tris-HCl, pH 7.5, 0.1 mm MnCl_2_, and 20 mm KCl buffer, and the samples were incubated at 37 °C for 2 h. Reactions were stopped as described above. Conversions were analyzed by spotting 1 μl of each reaction on a PEI-cellulose F TLC plate (Merck Millipore). The nucleotides were separated using a 1:1.5 (v/v) saturated NH_4_SO_4_, 1.5 m KH_2_PO_4_, pH 3.6 buffer, and visualized with a FLA 7000 Typhoon PhosphorImager.

##### In Vitro GdpP and Pde2 Enzyme Activity Assays

GdpP and Pde2 activity assays were performed using 50 mm Tris-HCl, pH 7.5, 0.1 mm MnCl_2_, and 20 mm KCl buffer, and incubated at 37 °C. Reactions were stopped at the indicated times by heating the samples to 95 °C for 10 min and subsequently removing the proteins by centrifugation at 13,000 rpm for 5 min. The activity of Pde2 was first assessed through hydrolysis reactions using ATP, c-di-AMP, and pApA as substrates. 100 μm concentrations of each substrate spiked with a low nm concentration of radiolabeled substrate were incubated with 1 μm GdpP or Pde2 for 2 h. This was performed twice with two separate preparations of purified Pde2 (four times in total). Time course experiments were initiated by incubating 200 μm c-di-AMP or pApA (again spiked with a low concentration of the respective radiolabeled substrate) with 4 nm Pde2 for 1 h. 5 μl samples were removed from each reaction at times 0, 2.5, 5, 10, 15, 20, 30, and 60 min. This was performed twice with two separate preparations of purified Pde2 (four times in total). GdpP inhibition assays were performed by incubating 50 μm c-di-AMP (spiked with radiolabeled c-di-AMP) with 0.3 μm GdpP in the presence of pApA (Biolog) ranging from 0 to 0.6 mm for 1 h. Pde2 inhibition assays were performed by incubating 200 μm pApA (spiked with a low concentration of radiolabeled pApA) with 4 nm Pde2 in the presence of ppGpp (tebu-bio) ranging from 0 to 0.8 mm. Inhibition assays were performed in triplicate. After heat inactivation and centrifugation, 1 μl from each reaction was spotted onto PEI-cellulose F TLC plates (Merck Millipore) and run in 1:1.5 (v/v) saturated NH_4_SO_4_ and 1.5 m KH_2_PO_4_, pH 3.6 buffer. Conversions were visualized using a FLA 7000 Typhoon phosphorimager and quantified using the ImageQuantTL software. Values were calculated based on an average from at least three individual experiments and plotted with standard deviations.

##### Acid Susceptibility Assay and Generation of Suppressor Strains

The indicated *S. aureus* strains were grown overnight at 37 °C in 5 ml of TSB. Cultures were normalized to an *A*_600_ of 5, 10-fold serial dilutions prepared, and 5 μl of the 10^−1^-10^−7^ dilutions were spotted onto TSA or TSA pH 4.5 plates containing 200 ng/ml Atet where indicated. To raise acid-resistant suppressor mutants, cultures of strain LAC*Δ*ybbR* were grown overnight in TSB, and 100 μl of 10^−1^ and 10^−2^ dilutions were spread onto TSA pH 4.5 plates. To confirm that the obtained colonies were indeed acid-resistant *ybbR* suppressor strains, several colonies were picked and grown overnight in TSB, and the next day 5 μl of 10^−1^-10^−6^ dilutions were spotted onto TSA pH 4.5 plates. A strain that was able to plate at the 10^−6^ dilution was considered a *bona fide* suppressor strain, and in this manner strains LAC*Δ*ybbR*-S1 to S5 and LAC*Δ*ybbR*-S7 to S11 were generated.

##### Whole Genome Sequencing

WT LAC*, LAC*Δ*ybbR*, and LAC*Δ*ybbR* suppressor strains were cultured overnight at 37 °C, cells were harvested, and genomic DNA was extracted. Genome sequencing for the *S. aureus* strain LAC* was performed by MicrobesNG using an Illumina Hi-Seq platform and a 250-bp paired end read kit. All other strains were sequenced at the Department of Microbiology and Immunobiology at the Harvard Medical School using an Illumina Mi-Seq platform and a 150-bp paired end read kit. Sequence analysis was performed using the CLC Genomics work bench software package. First the LAC* reads were aligned against the published USA300 FPR3757 genome sequence (RefSeq accession number NC_007793.1) and assembled into a reference contig, and the USA300 FPR3757 annotation and base numbering was transferred onto the LAC* sequence. Next, the LAC*Δ*ybbR* and LAC*Δ*ybbR* suppressor strain Illumina reads were mapped onto the assembled LAC* sequence, and high frequency (>65%) and good quality base changes were identified using the CLC Genomics Workbench software package; the data are summarized in Table 1. The Illumina short reads are deposited in the European Nucleotide Archive under accession numbers PRJEB14759 for strain LAC* and PRJEB14723 for all other strains.

##### c-di-AMP Quantification by Competitive Enzyme-linked Immunosorbent assay (ELISA)

5 ml of TSB was inoculated with a single colony of WT LAC* or the indicated *S. aureus* mutant strains and incubated for 4 h at 37 °C with aeration. Next, the cultures were diluted to an *A*_600_ of 0.05 in 5 ml of TSB and incubated overnight under the same conditions. For growth in acidified medium (pH 4.5 and pH 4.25), 5 ml of TSB was inoculated with single colonies of WT and mutant strains and incubated overnight at 37 °C with aeration. Cultures were diluted to an *A*_600_ of 0.05 in 5 ml of acidified TSB (and TSB pH 7.3 as a control) and grown to an *A*_600_ of 0.7. Cells were harvested from overnight or exponential phase cultures and washed 3 times with 1 ml of PBS, pH 7.4. Pellets were suspended in 1 ml of lysis buffer (50 mm Tris, pH 8, containing 20 μg of lysostaphin) and then incubated at 37 °C for 15 min. Lysates were added to 0.5-ml glass beads and lysed using a Fast-Prep instrument (FP120, MP Biomedicals, LLC) at setting 6 for 30 s. This was repeated twice, placing the samples on ice for 2 min between runs. Glass beads were removed by centrifugation at 17,000 × *g* at 4 °C for 5 min, and the cleared cell lysate was transferred to a fresh tube. Lysates were boiled for 10 min, debris were removed by centrifugation, and the supernatants were transferred to a new tube. The protein concentration of each sample was determined using the BCA kit (Pierce), and cell lysates were normalized to a concentration ranging from 0.3 to 0.75 mg/ml. The ELISAs were performed as previously described (61). Briefly, the wells of a 96-well NUNC Maxisorp plate were coated with 100 μl of a 10 μg/ml CabP protein solution in coating buffer (50 mm Na_2_CO_3_, 50 mm NaHCO_3_, pH 9.6), and plates were incubated overnight at 4 °C. Wells were washed 3 times with PBS, pH 7.4, containing 0.05% Tween 20 (PBST) and blocked with 5% BSA in PBS. Next the control samples and *S. aureus* extracts (in triplicate) were added to the plate. To prepare the control samples, c-di-AMP (BioLog) was diluted 2-fold in 50 mm Tris, pH 8 buffer, from 200 to 3.125 nm. The same volume of biotinylated c-di-AMP (BioLog) was added to each sample to give a final concentration of 25 nm. Next, 100 μl of these control and *S. aureus* extracts samples were added to the coated ELISA plate. 100 μl of lysis buffer only was added as a background reading control, and the plate was then incubated at room temperature for 2 h. After washing the wells 3 times with 200 μl of PBST, 100 μl of the Pierce^TM^ High Sensitivity Streptavidin solution (ThermoFisher Scientific) diluted 1:5,000 or 1:10,000 in PBS was added to the wells, and the plate was incubated at room temperature for 1 h. Wells were washed again 3 times with 200 μl of PBST. 10 mg of *o*-phenylenediamine dihydrochloride (Sigma) was dissolved in 20 ml of citrate buffer, pH 5, containing 20 μl of H_2_O_2_, and 100 μl was added as substrate to each well. The plate was incubated at room temperature for 30 min, and the reaction was stopped by the addition of 100 μl of 2 m H_2_SO_4_. The *A*_490_ was measured on a plate reader, buffer only readings were subtracted from controls, and buffer/lysostaphin readings were subtracted from samples. The absorbance readings for the c-di-AMP standard control samples were used to generate a second-order polynomial calibration curve, and the obtained quadratic equation was used to determine the c-di-AMP levels in the *S. aureus* extracts. nm of c-di-AMP were converted into ng of c-di-AMP/mg protein, and average values and standard deviations from three samples plotted. A two-tailed two sample equal variance Student's *t* test was used to determine statistically significant differences between c-di-AMP levels in WT *versus* mutant strains.

## Author Contributions

L. B. and A. G. designed the study and wrote the paper. L. B., M. S. Z., C. F. S., and A. G. performed the experiments. V. K. designed the nucleotide mass spectrometry and HPLC-UV experiments, and all authors analyzed the results and approved the final version of the manuscript.

## Footnotes

4 The abbreviations used are:

MRSA: methicillin-resistant *S. aureus*
c-di-GMP: cyclic di-guanine monophosphate
(p)ppGpp: guanosine tetraphosphate and pentaphosphate
c-di-AMP: cyclic di-adenosine monophosphate
DAC: diadenylate cyclase
pApA: 5′-phosphadenylyl-adenosine
PDE: phosphodiesterase
pAp: 3′-phosphoadenosine-5′-phosphate
TSB: tryptic soya broth
MIC: minimal inhibitory concentration
Atet: anhydrotetracyline
compl.: complementation strain
SOE: splice overlap extension.
